# Quartz Crystal Microbalance Application and *In Silico* Studies to Characterize the Interaction of Bovine Serum Albumin with Plasma Polymerized Pyrrole Surfaces: Implications for the Development of Biomaterials

**DOI:** 10.1021/acs.langmuir.3c00308

**Published:** 2023-08-01

**Authors:** Iris N. Serratos, Alberto S. Luviano, Cesar Millan-Pacheco, Juan Morales-Corona, Estephanny Jocelyn Alvarado Muñoz, José Campos-Terán, Roberto Olayo

**Affiliations:** † Departamento de Química, Universidad Autónoma Metropolitana-Iztapalapa, Ciudad de México 09340, México; ‡ Laboratorio de Biofisicoquímica, Departamento de Fisicoquímica, Facultad de Química, Universidad Nacional Autónoma de México, Ciudad de México 04510, México; ⊥ Facultad de Farmacia, Universidad Autónoma del Estado de Morelos, Morelos 62209, México; ∥ Departamento de Física, Universidad Autónoma Metropolitana-Iztapalapa, Ciudad de México 09340, México; # Departamento de Ingeniería Eléctrica, Universidad Autónoma Metropolitana-Iztapalapa, Ciudad de México 09340, México; □ Departamento de Procesos y Tecnología, Universidad Autónoma Metropolitana-Cuajimalpa, Ciudad de México 05348, México

## Abstract

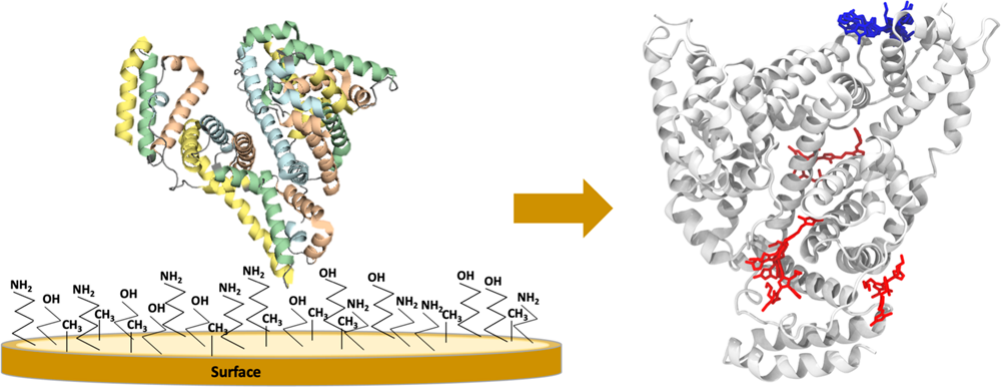

Plasma polymerized pyrrole/iodine (PPPy/I) microparticles and bovine serum albumin (BSA) protein have shown interesting results in experimental models for the treatment of traumatic spinal cord injury. By studying the interaction between BSA and PPPy/I by a quartz crystal microbalance (QCM) and docking, we obtained important results to elucidate possible cellular interactions and promote the use of these polymers as biomaterials. These measurements were also used to characterize the adsorption process using an equilibrium constant. In addition, atomic force microscopy (AFM) was used to obtain images of the QCM surface sensors before and after BSA adsorption. Furthermore, we carried out molecular dynamics simulations and molecular docking to characterize the molecular recognition between BSA and the previously reported PPPy/I structure. For this study, we used two combinatorial models that have not been tested. Thus, we could determine the electrostatic (Δ*G*_ele_) and nonelectrostatic (Δ*G*_nonelec_) components of the free binding energy (Δ*G*_b_). We demonstrated that BSA is adsorbed on PPPy/I with an adsorption constant of *K* = 24.35 μ^–1^ indicating high affinity. This observation combined with molecular docking and binding free energy calculations showed that the interaction between BSA and both combinatorial models of the PPPy structure is spontaneous.

## Introduction

Biomaterials derived from the pyrrole molecular structure have recently been developed, and these materials present electrostatic properties and amine groups that promote interaction with proteins and favor cellular anchors due to their chemical diversity. In this context, polypyrrole particles synthesized by plasma with dopants have shown biocompatibility with spinal and cord tissues. Álvarez-Mejia et al. demonstrated that when a suspension of microparticles of plasma polymerized pyrrole/iodine (PPPy/I) was injected into the injured spinal cord site in rats, motor function was recovered,^1^ and films of PPPy/I provided good results for the evolution of injured spinal cord in rhesus monkeys.^2^ Notably, PPPy/I has also been tested in new *in vitro* techniques that optimize the culture of nerve, motor neurons, and muscle cells.^3,4^ In fact, recent evidence collected by our group demonstrated the effect of a combined treatment of bovine serum albumin (BSA) with PPPy/I on motor recovery after traumatic spinal cord injury (TSCI) in rats.^5,6^ The possibility that BSA could engage in direct physicochemical interactions with the microparticles of PPPy/I is an interesting alternative explanation for the results observed in the spinal cord of rats.

**Figure 1:**
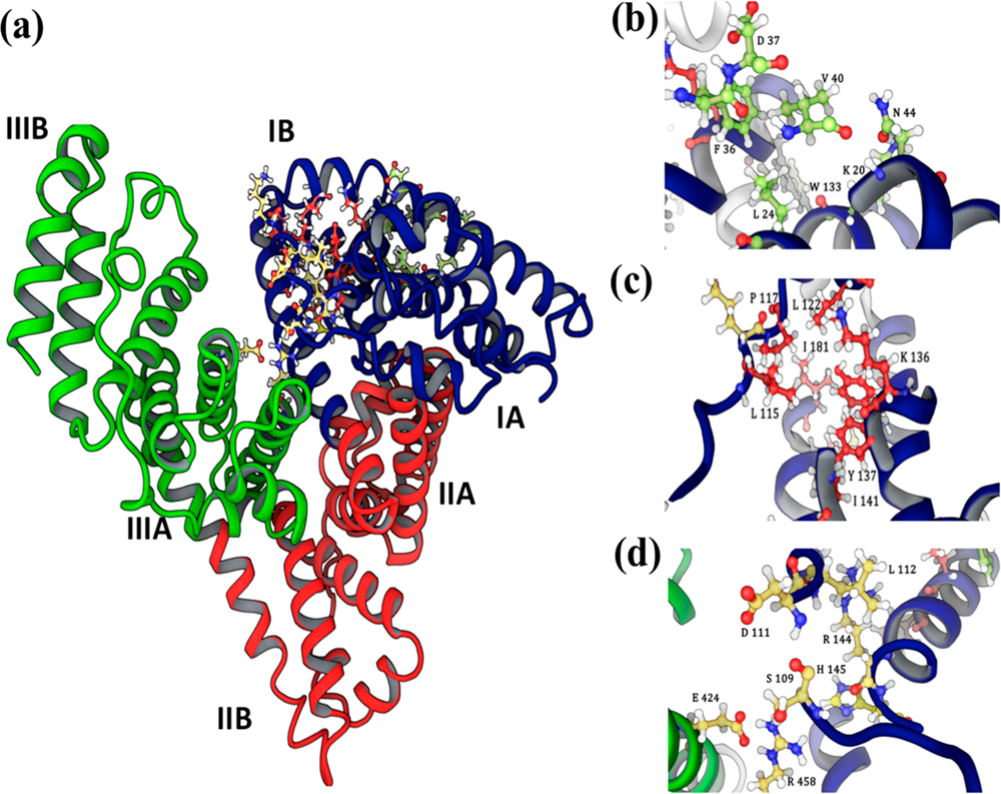
(a) Crystal structure of serum albumin protein (BSA) (PDB ID 3V03) showing three domains. BSA is composed of hydrophobic cavities: (b) Cavity 1, (c) Cavity 2, and (d) Cavity 3. The 3D structure was prepared with Maestro 2019-4 program.

BSA has been characterized as a globular protein that weighs 66 kDa. In addition, BSA consists of 583 amino acids in three homologous α-helical domains, and each domain is divided into two subdomains (A and B). BSA has an average hydrodynamic diameter of 7.12 nm, a gyration radius of 2.76 nm and a partial specific volume of 125 nm.^3,7,8^ According to the literature, BSA has an isoelectric point at pH in the range of ∼4.8–5.0, whereas at pH 7.2, it shows a negative surface charge of ∼18 mV. It is important to mention that the Zeta potential of BSA has been determined at different salt concentrations and pH providing insights into the importance of the electrostatic interactions in its adsorption process to surfaces.^9−11^ Moreover, there is an increasing number of studies that involve the adsorption of proteins on biomaterials using BSA,^12,13^ integrins,^14^ and lysozyme,^15−17^ among others. Additionally, several studies have demonstrated that BSA is a stabilizing agent for nanoparticles in general.^13,18−21^ Wu et al. showed a detailed study of binding of ligands to the hydrophobic cavities of BSA by molecular docking. Cavities 1 and 2 were found in domain I, although the first cavity is on the BSA surface. Cavity 3 is found between domains I (subdomain IIA) and III (subdomain IIIA) (see Figure 1).^22^ The 3D structure was visualized with the Maestro 2019-4 program.^23^

**Figure 2:**
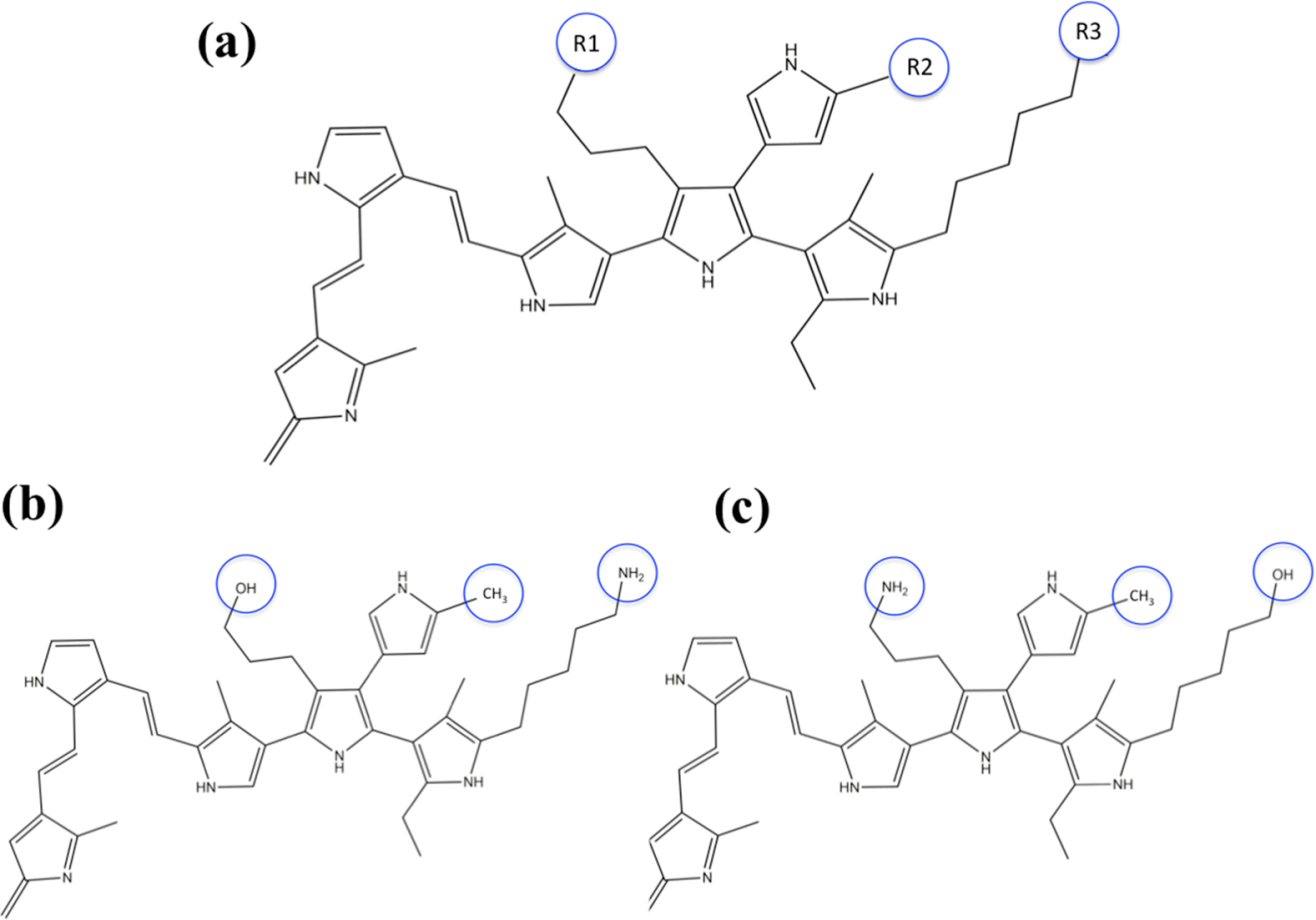
(a) Chemical structure of modified PPPy based on that reported by Kumar et al., indicating the R1, R2, and R3 ramifications in 2D. (b) The position of R1 = OH, R2 = CH_3_, R3 = NH_2_ and (c) R1 = NH_2_, R2 = CH_3_, R3 = OH. Each structure was built up with the program Marvin version 17.21.0, ChemAxon (https://www.chemaxon.com).

Likewise, polypyrrole is a well-known polymer with excellent biocompatibility and therefore has been applied in several biomedical studies.^24,25^ Polypyrrole is an organic polymer consisting of pyrrole units, which are heterocyclic aromatic structures composed of four carbon atoms and one nitrogen atom. Previous studies by Kumar et al.^26^ proposed a PPPy structure which was reported from IR spectra, and broad bands were found in the polymer spectra from NH bending, C=N stretching, and C=C stretching in amine, imine, and alkene units. The researchers also found that functional groups can be introduced at the R1, R2, and R3 positions on the pyrrole monomer.^26^ It is important to mention that this structure does not contain iodine. This PPPy structure was modified in R3 by adding carbon atoms to lengthen slightly the chain, since in this position, it would present more freedom of movement and could favor the union with proteins (Figure 2a). In this work, we utilized this modified PPPy structure as a template to carry out interaction studies with some proteins through docking assays and binding energy calculations.

In this regard, our group has recently obtained preliminary evidence for molecular interactions in the hydrophobic cavities of BSA; this evidence was obtained from two PPPy combinatorial models through a computational study to validate the interactions within each pocket. In the first model, different functional groups, such as methyl (CH_3_), hydroxyl (OH), and amino (NH_2_), were varied in the ramifications of the modified PPPy structure (R1, R2 and R3). In the second model, the nitrile (C≡N), OH and NH_2_ functional groups were varied in R1, R2, and R3. These functional groups were considered because they are present in the synthesis of PPPy. It was determined that the first model favored binding between BSA and the modified PPPy structure, in which the positions of the functional groups were R1 = OH, R2 = CH_3_, and R3 = NH_2_. Of the three BSA cavities, favorable interactions were established at cavity 1, which was reflected in the binding energy (Δ*G*_b_), in which hydrophobic interactions predominated with respect to electrostatic interactions.^27^ This study was followed by a second report, which was based on the interaction on the BSA surface and modified PPPy structure varying the position of OH, CH_3_ and NH_2_ functional groups in R1, R2, and R3 by docking studies. These results suggested that NH_2_ and OH must be in R1 and R3 to favor electrostatic interactions.^28^ Notably, the following combinations have not yet been tested in the modified PPPy structure: R1 = OH, R2 = CH_3_, and R3 = NH_2_ (Figure 2b) and R1 = NH_2_, R2 = CH_3_, and R3 = OH (Figure 2c), in which CH_3_ remains fixed in R2 in both cases. Structures were built up with the program Marvin version 17.21.0, ChemAxon (https://www.chemaxon.com).^29^

**Figure 3:**
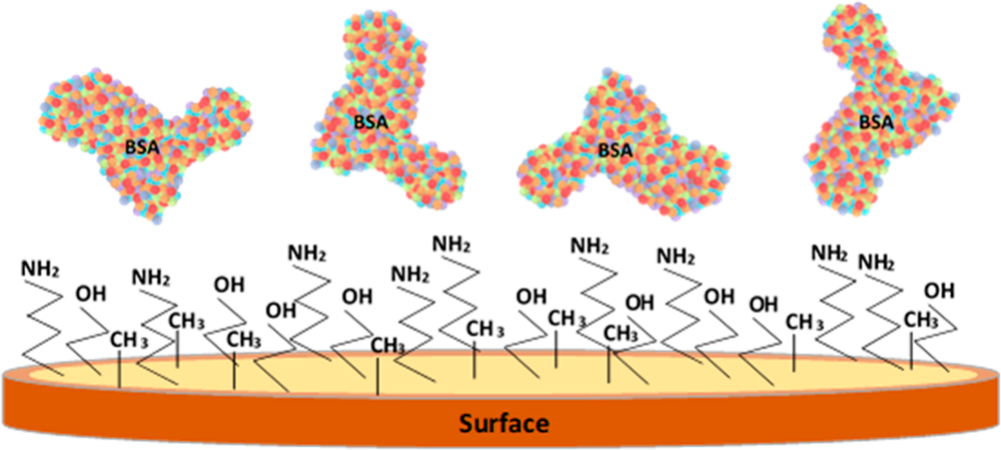
Schematic representation of BSA adsorption to the PPPy network.

Therefore, we were especially interested in characterizing these combinations in the process of BSA adsorption on a PPPy surface network and considered both experimental (Figure 3) and *in silico* studies.

In this work, we investigated the direct interaction between BSA and PPPy/I through performing measurements on a quartz crystal microbalance (QCM) using gold sensors, which were covered with a network of PPPy/I. Since information about the adsorption process can be obtained through QCM, we also employed atomic force microscopy (AFM) to have visual structural evidence and characterize the adsorbed protein.^30,31^

On this PPPy/I surface we considered that it is present an average characteristic structure of PPPy/I that expresses different and mobile functional groups and an important chemical diversity to interact with BSA. In this sense, we used the modified PPPy structure based on in the structure determined by Kumar et al.^26^ as an “average characteristic surface” in order to study its interaction with BSA, thus obtaining a simplified model, which will allow us to study this recognition at the molecular level.

Hence, we simulated the interaction between BSA and the modified PPPy structure based on that determined by Kumar et al.^26^ to study the two combinations mentioned above that were not tested, in which CH_3_ remained in position R2. These studies are complementary to previous reports by Fabela et al.^5,6^ that utilized an experimental model where BSA-PPPy/I treatment in TSCI rats favored motor function recovery and they observed that BSA interacted with PPPy/I. In general, protein–polymer interactions are an important key in the design of scaffolds and therapies in spinal cord cells. In this work, our findings confirm the direct interaction between BSA and PPPy/I by QCM (determining an adsorption constant) and computational studies. Both approaches provide a physicochemical explanation of the molecular recognition phenomena.

## Experimental and Computational Methods

### Materials

Pyrrole monomer, iodine, and bovine serum albumin (BSA) lyophilized powder (05470) were purchased from Sigma-Aldrich Co. (St. Louis, MO) and used without further purification. All aqueous samples were prepared using ultrapure water obtained from a Millipore system (18.2 MΩ) at a controlled temperature of 25 °C.

### Dynamic Light Scattering of BSA Solution

BSA was prepared in phosphate buffer (pH 7.2) at 2 μM and heated at 30 °C for 30 min, and each solution was filtered before use with a 0.2 μm Whatman filter to remove the aggregated protein. BSA stock solutions were used immediately after preparation. The particle size distribution was measured using a dynamic light scattering (DLS) instrument (Zetaseizer Nano-ZS, Malvern Instruments Ltd., Malvern, UK) with a 660 nm He–Ne laser and configured in a backscattering position (173°). Through the equipment software, the average hydrodynamic diameter was calculated before each QCM experiment, using a nonnegative least-squares analysis. In our case, we obtained a z-average diameter value of approximately 8.8 nm (considering only the first peak, which represents the monomeric proteins in solution, see Figure S1 in the Supporting Information), which is close to the long axis of the BSA molecular conformation.^32^

### Preparation of PPPy/I-Coated Gold Surfaces

**Figure 4:**
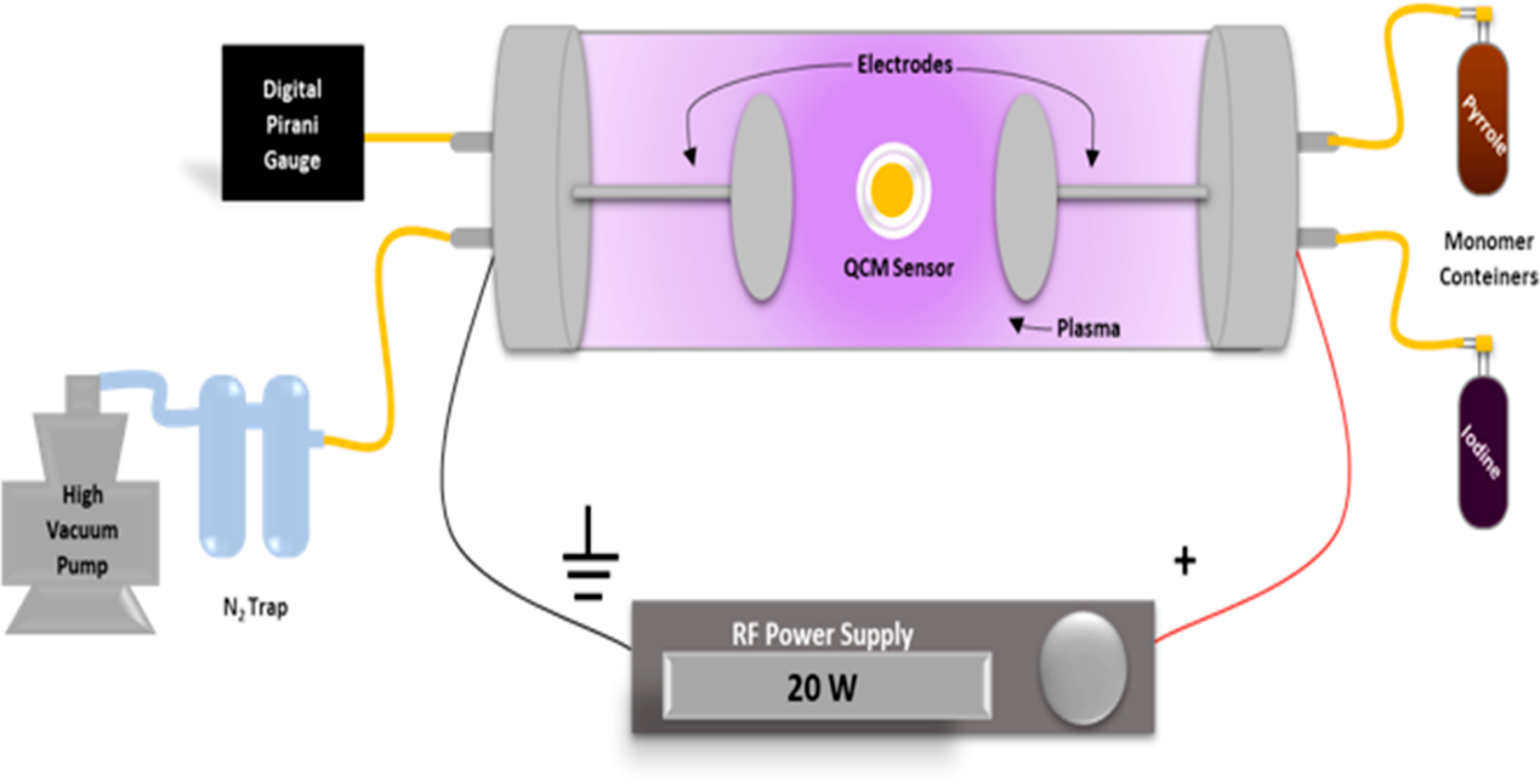
Schematic representation of the plasma reactor used for the deposition of the PPPy/I film on the QCM sensors.

QCM sensors, AT-cut quartz crystals with a fundamental resonance frequency of ∼5 MHz, were purchased from Q-Sense (Biolin Scientific, Gothenburg, Sweden) with a bare gold coating layer (QSX 301). The gold sensors were cleaned before use with ultrapure water and chloroform and dried under a nitrogen stream. Likewise, the polypyrrole film was deposited on the QCM sensors in a cylindrical glass reactor coupled to a high vacuum pump (Alcatel Pascal 2015 C1, Ideal Vacuum Products, Albuquerque, USA) generating a 0.55 Torr vacuum and to two J. Young vacuum valves (6 mm output) with pyrrole monomers, and iodine was introduced to the reactor. On each side of the reactor, two electrodes made of stainless-steel bars and plates are located. The electrodes were directly connected to a 13.56 MHz radio frequency power supply (Cesar 1500, Dressler Advanced Energy, Metzingen, Germany) to initiate glow discharges. The system was supplied with 20 W of power, with a total reaction time of 3 min, 2 min of pyrrole, and 1 min of iodine (Figure 4). Physicochemical characterization of PPPy/I films at surfaces produced by this method has been done before^33,34^ (Figures S2 and S3 in the Supporting Information). In general, the results obtained confirm the presence of the functional groups structure proposed for a film by Kumar et al.^26^

### Atomic Force Microscopy (AFM)

AFM was used to obtain images of the QCM sensor surface before (gold and PPPy/I films) and after BSA adsorption. The sensors were attached to a magnetic sample holder, and AFM imaging was carried out with a NaioAFM device (Nanosurf AG, Switzerland) using an intermittent contact mode cantilever (Tap190AI-G, BudgetSensors). The images were collected in different scan sizes from 2 to 50 μm at a scan rate of 1 Hz. Standard imaging procedures and setting parameters were used, and height and phase images were recorded.

### QCM Theory and Experiment

BSA adsorption was measured using a QCM system (KSV Instruments, Ltd., Finland) equipped with a temperature-controlled chamber and a peristaltic pump. The QCM is a sensitive mass sensor per unit area that measures the change in the fundamental resonance frequency of a quartz crystal sensor due to the adsorption of a material on its surface.^35^ One way to describe this adsorption process is with the Sauerbrey equation,^36^ which relates the frequency shift (Δ*f*) with surface coverage (ΔΓ) over the QCM crystal as follows:
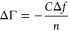
1where *C* = −17.7 ng cm^–2^ Hz^–1^ is a calibration constant that involves the resonance frequency of the unloaded resonator, the density and shear modulus of the quartz, and the electrode area of the sensor, and *n* is the overtone order, which also normalizes the equation. The results shown in this study were calculated using the third overtone of a 5 MHz fundamental resonance frequency gold sensor (Biolin Scientific, Finland). The use of the Sauerbrey equation assumes that the adsorbed layer is rigid or that the adsorbed film is thin enough to perfectly couple with the resonator. When the adsorbed material forms a viscoelastic layer, the adsorbed mass should be higher than the adsorbed value calculated by Sauerbrey’s equation due to, for example, water adsorption. This viscoelastic behavior produces dissipative energy, and in the typical QCM geometry, it can be calculated as follows:
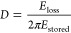
2where *E*_loss_ is the energy dissipated during one oscillation period and *E*_stored_ is the stored energy in the system. When this value is lower than 2 × 10^–6^ units, the system has no viscoelastic contribution. All measurements of BSA adsorption performed in this work revealed a value of *D* lower than this limit; therefore, we used Sauerbrey’s equation to calculate the adsorbed mass per unit area.

Before every measurement, the PPPy/I sensors were cleansed with water and dried under a nitrogen stream. The sensor was placed inside the QCM chamber, and then a phosphate solution (20 mM, pH 7.2) was injected in a 1 mL/min flow for at least 10 min until a stable baseline signal was obtained. From this point, BSA solutions were injected into the sensor from lower to higher concentrations until they reached a stable frequency at each concentration. Between each concentration, a rinsing phosphate buffer (20 mM at pH 7.2) solution was injected to detach any loosely adsorbed material. This procedure was also carried out at the end of the highest concentration injection. All the data obtained were determined in triplicate, and the error bars are calculated as the standard deviation. The temperature of each experiment was controlled with the QCM system at 25 °C.

### *In Silico* Studies

#### Molecular Dynamics of BSA

Bovine serum albumin protein from *Bos taurus* was used in this study (PDB^37^ ID: 4F5S). Crystallographic water molecules were kept before the system was solvated. A cubic water box was constructed with a distance of 10 Å from any protein edge. The KCl concentration was set at 0.15 M. System was constructed using the CHARMM-GUI web server,^38,39^ and molecular dynamics were handled using the input files generated by the server. Molecular dynamics simulations were run for 50 ns using NAMD 2.12.^40^ Cluster analysis was realized using Simulaid software^41^ with the maximum neighborhood method as implemented with a cutoff of 2 Å over the last 20 ns. The center of the most populated cluster found was used to perform molecular docking with a PPPy structure.

The modified PPPy chemical structure was constructed (Figure 2a) using ChemAxon^29^ based on the structure proposed for a film by Kumar et al.^26^ In this work, we present two combinatorial models of the PPPy structure varying in one case R1 = OH, R2 = CH_3_, and R3 = NH_2_ (Figure 2b) and in the other R1 = NH_2_, R2 = CH_3_, and R3 = OH (Figure 2c), where CH_3_ remains fixed in R2.

#### Interaction of the PPPy Structure on the BSA Surface by Molecular Docking

Molecular blind docking was realized with AutoDock Vina^42^ over all protein structures as implemented on Chimera UCSF,^43^ and 1000 independent molecular docking experiments were conducted, keeping the protein rigid and the ligand flexible. The final structures were analyzed by RMSD to obtain the most repetitive structure. All figures and interaction maps were made using Maestro from Schrodinger.^23^

#### Binding Energy: Computational Determinations

To determine the free binding energy (Δ*G*_b_), we used Nathan Baker’s methodology,^44^ which was used in previous reports to mainly study receptor–ligand interactions, and the results correlated very well with the experimental trend.^45−48^ In this work, to calculate Δ*G*_b_ for the poses of each combinatorial model of the PPPy structure on the BSA surface, we considered the electrostatic and nonelectrostatic components as described by Baker et al:^44^
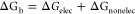
3Here, the electrostatic energy is divided mainly into two components, solvation and Coulombic energy, as follows:
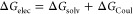
4where Δ*G*_solv_ represents the computational determination for solvation energies and Δ*G*_Coul_ represents the Coulombic energies of the complex and free species (BSA and PPPy structure). Both components were calculated using the Adaptive Poisson–Boltzmann Solver (APBS) program.^44^ Parameters for BSA were taken from the PDB 2PQR server.^49^ Ionic radii and atomic charges were assigned from the force field CHARMM.^50^ PROPKA was used to assign the protonation state of ionizable residues under physiological pH according to experimental conditions.^51^ PPPy structure atomic charges were taken from the molecular docking carried out by AutoDock Vina.^42^

The nonelectrostatic energy (Δ*G*_nonelec_) was estimated by multiplying the solvent-accessible surface area change (ΔSASA) by an interfacial tension coefficient as follows (γ = 5 cal mol^–1^ Å^–2^):^52,53^
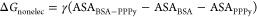
5The ASA calculations for complex and free species (BSA and PPPy structure) were performed using the Visual Molecular Dynamics (VMD) program,^54^ implying a probe radius of 1.4 Å. For a detailed analysis of the interactions at the binding site, we chose the conformation with the best Δ*G*_b_.

## Results and Discussion

### Sensor Characterization and Protein Adsorption

Protein adsorption experiments on hydrophobic substrates have shown that proteins, such as human and bovine serum albumin (HSA, BSA), generally change conformation by favorably exposing their hydrophobic groups to increase the hydrophobic interaction with the surface, leading to stronger protein adsorption.^55^ Since clean gold surfaces can be considered hydrophilic, we expect this type of protein (BSA in our case) to exhibit lower adsorption on these surfaces.^56^ Additionally, the coated PPPy/I sensors can be considered hydrophilic.^5^

**Figure 5:**
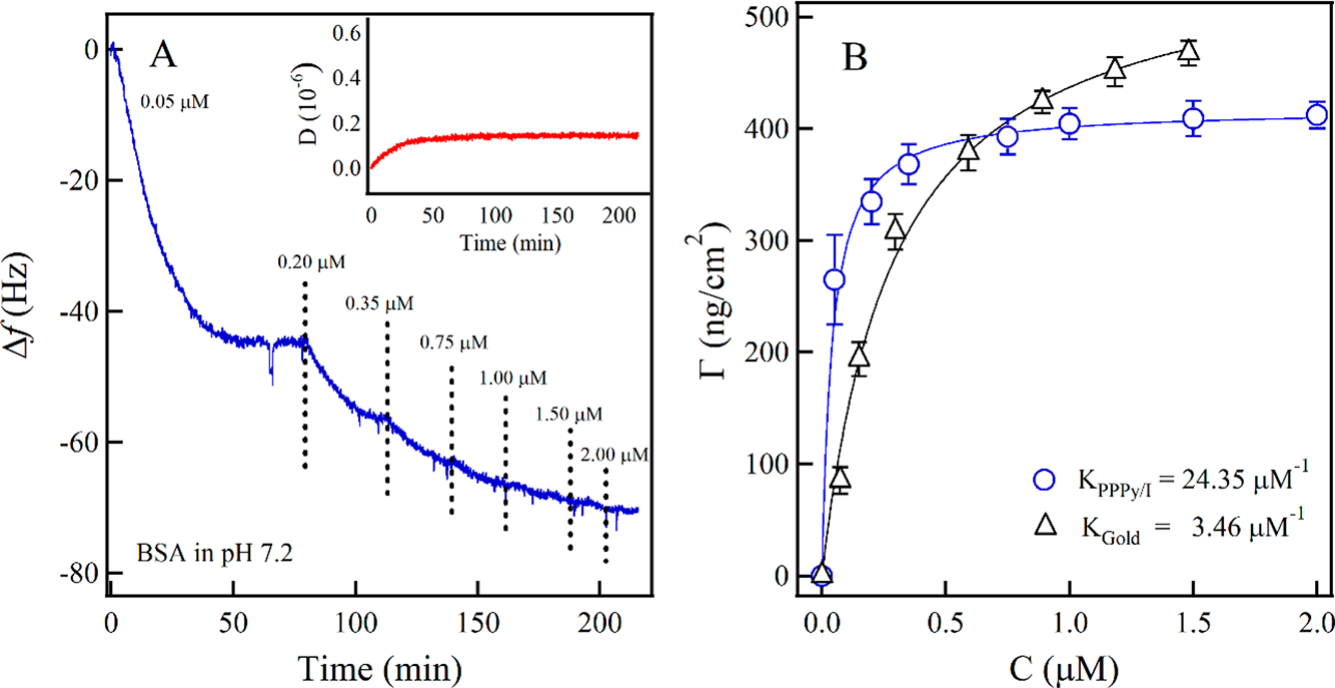
(A) Frequency change on the PPPy/I sensor is due to the adsorption of BSA protein at different concentrations. Large protein uptake occurs at lower concentrations. The inset shows how the dissipation has a low value; therefore, the adsorbed mass of the layer can be measured using the Sauerbrey equation. (B) Langmuir isotherm model uses the values of the frequency change limit for each concentration for BSA adsorption on bare gold and PPPy/I-coated gold surfaces.

Figure 5A shows the adsorption of BSA on the PPPy/I-coated surface. We observe a high increase in the frequency change with the lower, and first, injected concentration. The following injections, although presenting a lower frequency change, indicate that the protein continues adhering to the substrate until saturation adsorption is found. On the first injection (*C* = 0.05 μM), the frequency change is close to 45 Hz. Compared with experiments performed on the gold surface using HSA, in which the frequency reaches a value of ∼16 Hz with a concentration of *C* = 0.075 μM,^57^ the difference is almost 3 times that indicates a higher affinity of BSA for PPPy/I surfaces.

Since the values of dissipation obtained (Figure 5A inset) are low and constant, we can use the Sauerbrey equation to calculate the surface coverage (eq 1), as shown in Figure 5B for bare gold and gold PPPy/I-coated surface. From eq 1, we observe that the surface coverage is directly proportional to the frequency change. Additionally, as seen in Figure 5B, we can distinguish the following regimes: the first one at low concentrations (below 0.5 μM), in which all values of coverage from the PPPy/I-coated surface are higher than those of the bare-gold surface; and the second one at high concentrations (above 0.5 μM), in which the coverage of the bare-gold surface becomes higher than that of the PPPy/I-coated surface. To further understand the behavior of the system, we fit our results by using a model that relates the occupancy sites on the sensor surface with the allocation of proteins. This configuration is modeled by the Langmuir equation as follows:
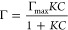
6where Γ is the surface coverage, *C* is the protein concentration, and *K* is the Langmuir equilibrium constant, which indicates the adsorption affinity to the surface. The higher coverage of BSA at lower concentrations over the PPPy/I sensor implies an increased affinity for this polymer film. In fact, by calculating this frequency change in every concentration step of both bare-gold and PPPy/I-coated surfaces, we obtained adsorption constants of *K*_Gold_ = 3.46 μM^–1^ and *K*_PPPy_ = 24.35 μM^–1^, respectively (Figure 5B). The adsorption constant value of the BSA to the PPPy/I-coated surface is two or three orders higher compared with that of other reported systems; for example, BSA has adsorption constants of 0.105, 0.058, and 0.018 μM^–1^, on ultrahigh molecular weight polyethylene, hydroxylapatite and stainless steel surfaces, respectively.^58−60^ This high adsorption constant can be explained by the favorable electrostatic interactions of the BSA protein with the PPPy surface. At pH 7.2, a high number of NH_2_ (located at R1 or R3 in the PPPy molecule, see Figure 2) are protonated, giving a partial positive charge to the surface, and since BSA protein at this same pH shows a negative charge, its attraction to the surface occurs at lower concentrations than in the bare gold sensor until the surface reaches saturation, at which the possible further aggregation is negligible (saturation in Figure 5B). On the other hand, for the bare-gold surfaces, proteins may continue populating the surface in a small quantity, and other factors could be involved, such as a possible hydrophobic effect.

**Figure 6:**
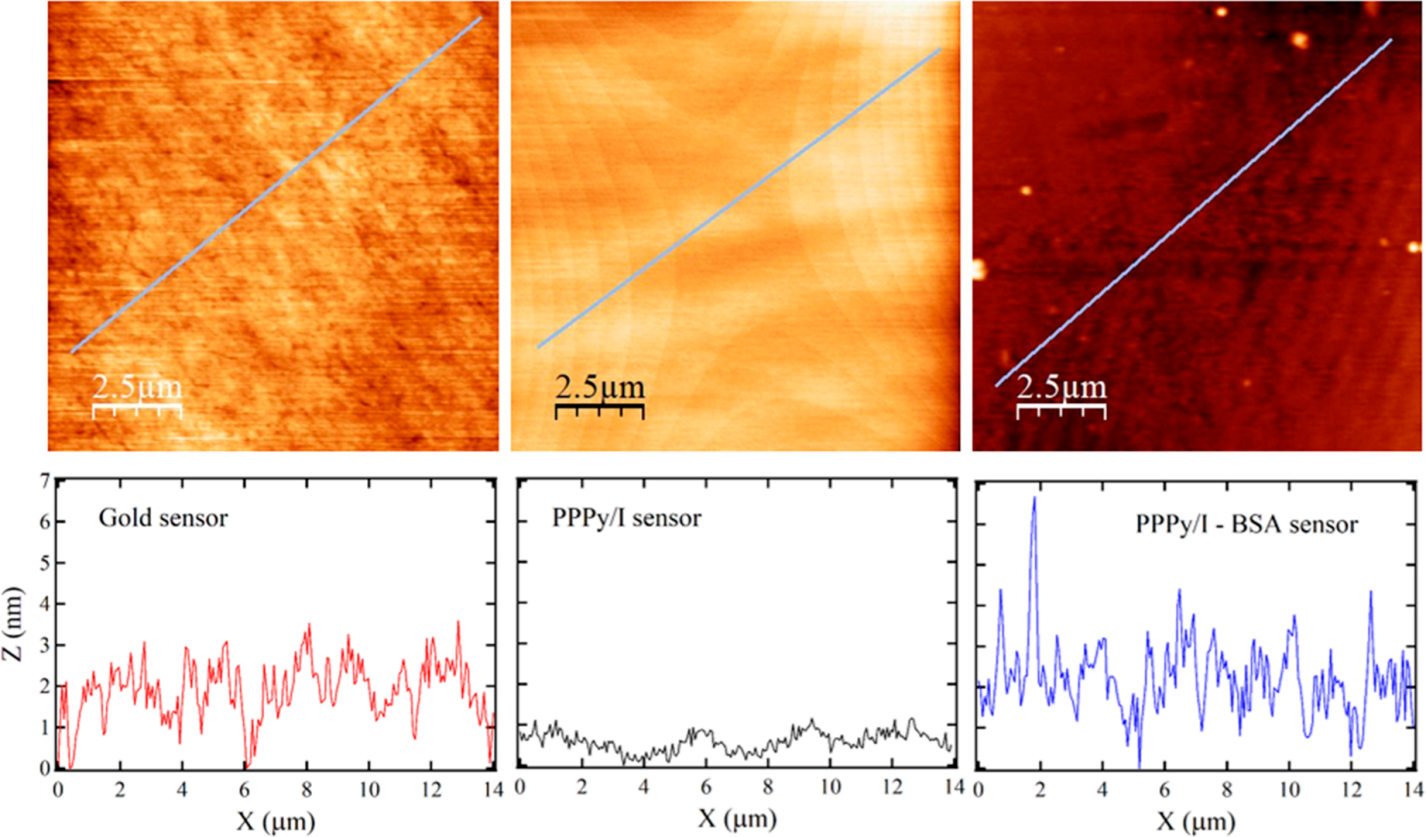
Representative images of (left) clean gold surface, (center) PPPy/I-coated surface using a plasma reactor, and (right) a PPPy/I-coated surface with BSA protein adsorbed using a QCM experiment. The three images are from the same sensor in each step. The images below show the roughness profile over the drafted line in each of the AFM images.

The surface topology of the sensors was also characterized by AFM. Figure 6 (left) shows an image of a clean gold QCM sensor with an average roughness value of 0.827 nm, which corresponds to the value given by the fabricant. The PPPy/I-coated sensor was also analyzed (Figure 6 center), and we can observe a pattern of curve lines that, at the shown resolution, may indicate that the PPPy/I deposition was forced to follow the electromagnetic field pattern produced by the electrodes on the plasma reactor (more AFM images of different regions and various magnifications sizes are shown in Figures S4 and S5 in the Supporting Information). The PPPy/I deposition method produces a smoother surface than that of the clean gold sensor with an average roughness value of 0.632 nm. Figure 6 (right) shows the surface of a PPPy/I-coated sensor after a BSA protein adsorption QCM experiment was performed. In this case, after the experiment was performed, the sensor was left to lose its water content through evaporation for 2 days. In addition, the roughness analysis showed an average value of 2.171 nm, although the surface looked homogeneous enough, despite a few aggregates. In principle, if two surfaces show the same water affinity but different topology, the surface that is more available (higher roughness) will adsorb more protein; in our case, both surfaces are hydrophilic but exhibit different roughness. However, the smoother surface still shows higher protein adsorption at the lower studied concentrations, demonstrating a higher affinity of BSA for the PPPy/I surface.

### Interaction between BSA Surface and Each Combinatorial Model of the PPPy Structure by Computational Studies

#### Molecular Docking Studies

We have demonstrated in the last section that BSA binds to a PPPy/I surface, but unfortunately, the binding site is unknown. To solve this aspect, we used the modified PPPy structure based on the determined by Kumar et al.^26^ as an “average characteristic surface” in order to study its interaction with BSA, thus obtaining a simplified model, which will allow us to study this recognition at the molecular level. We carried out docking studies on the surface of BSA and the PPPy structure (with two combinatorial models). Molecular docking studies are a common technique to corroborate or propose the ligand binding mode of a molecule with its therapeutic target.^33^ Previously, we carried out three independent 100 ns molecular dynamics simulations on HSA (PDB ID 5YB1) with a resolution of 2.62 Å (Figure S6 in the Supporting Information). These HSA results help us to validate the result presented for BSA, which mainly reports a high electrostatic interaction in its interaction with the combinatorial model R1 = OH, R2 = CH_3_, and R3 = NH_2_ compared to R1 = NH_2_, R2 = CH_3_, and R3 = OH (Tables S1, S2 and S3 in the Supporting Information).

**Figure 7:**
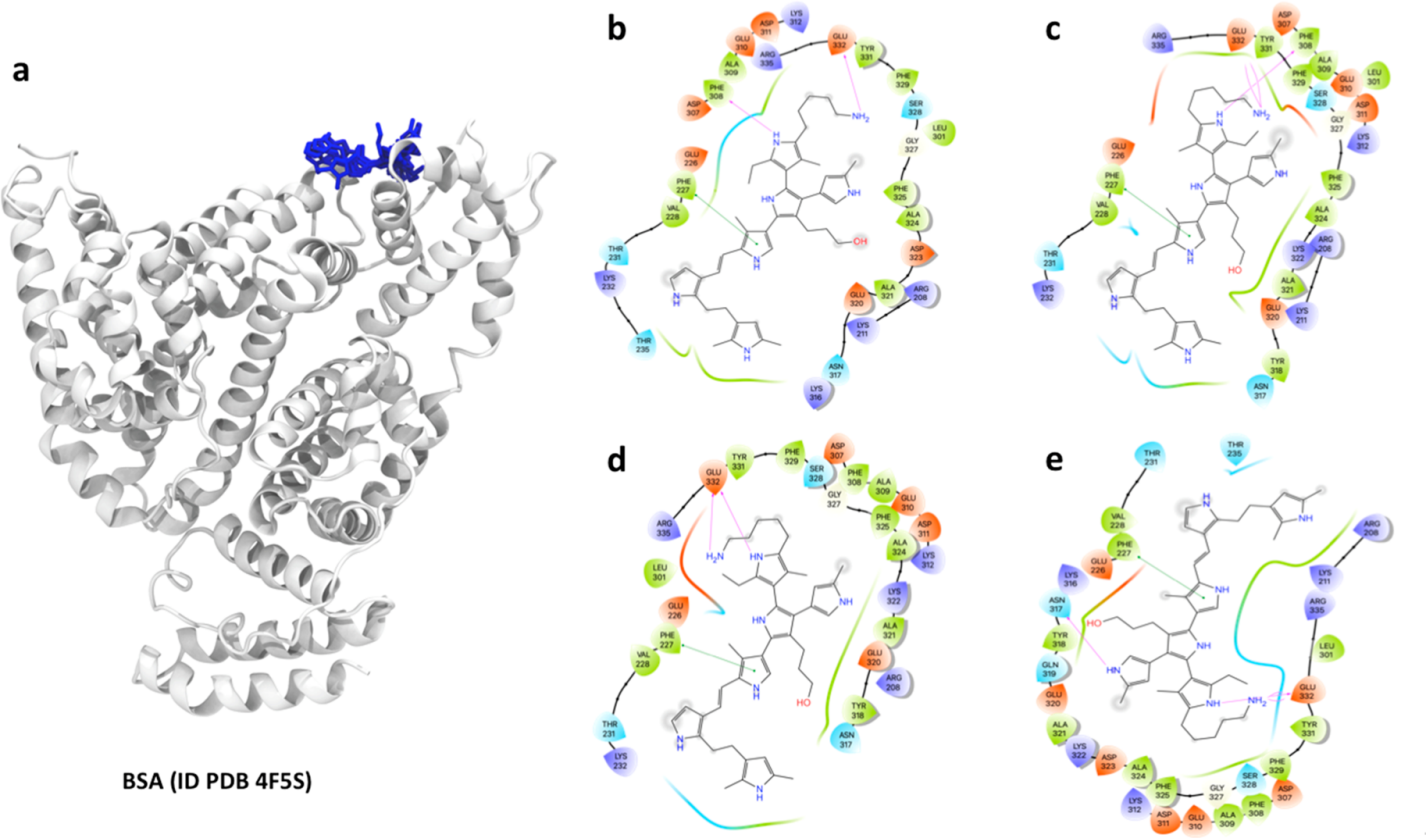
Best docked conformations for the complex of BSA with PPPy: (a) Binding modes of the combinatorial model R1 = OH, R2 = CH_3_, and R3 = NH_2_ (blue color) to BSA: Compounds were ordered by the most favorable binding energy of the most populated cluster of Group 1 according to Table 1 and were prepared with VMD:^54^ (b) Complex 1, (c) Complex 2, (c) Complex 3, and (d) Complex 4. Interaction maps were obtained with the Maestro 2019-4 program.

In this work, the albumin protein from *Bos taurus* (PDB ID: 4F5S) was relaxed from its crystallographic conformation with 50 ns of molecular simulation. Cluster analysis of the last 20 ns was conducted, and the center of the most populated cluster was used for molecular docking with the PPPy ligand. One thousand independent blind experiments were performed, and the four most populated clusters (hereafter named complexes 1 to 4) were analyzed to examine their interaction with PPPy. For detailed analysis of the interactions, we analyzed all possible poses according to the best Δ*G*_b,_ as shown in Table 1. Interaction sites and representations of the combinatorial model R1 = OH, R2 = CH_3_, and R3 = NH_2_ to BSA are shown in Figure 7.

**Table 1:** Δ*G*_b_ Values for BSA with the Combinatorial Model R1 = OH R2 = CH_3_, and R3 = NH_2_ of the PPPy Structure Determined at pH 7.2 by APBS^44^ and VMD1.9.1

Clustering analysis		Δ*G*_solv_ (kJ/mol)	Δ*G*_Coul_ (kJ/mol)	Δ*G*_nonelec_ (kJ/mol)	Δ*G*_b_^a^ (kJ/mol)
Group 1	Complex 1	77	–469	–22	–414
	Complex 2	86	–462	–22	–398
	Complex 3	105	–452	–22	–369
	Complex 4	131	–464	–23	–356

a Δ*G*_b_ = Δ*G*_solv_ + Δ*G*_Coul_ + Δ*G*_nonelec_ (2).

The docking results showed a specific site for the PPPy structure (combinatorial model R1 = OH, R2 = CH_3_, and R3 = NH_2_) on the surface of BSA (Figure 7a). Figure 7b shows Complex 1, in which a hydrogen bond was formed between the longest chain (R3 = NH_2_) of the PPPy structure and the COO^–^ group of Glu 332 of BSA (N–H···O, respectively). A second hydrogen bond was established between the NH of a pyrrole ring of the PPPy structure and the Phe 308 group. A π–π stacking interaction is presented between Phe 227 and the pyrrole ring of the PPPy structure. The chain (R1 = OH) of the PPPy structure caused an electrostatic environment with Arg 208, Glu 320 and Asp 323. The short chain (R = CH_3_) interacts with hydrophobic amino acids Phe 227 and Val 228. The interactions presented by Complex 2 (Figure 7c) are very similar to the previous case except that here, the longest chain (R3 = NH_2_) established two hydrogen bonds with Glu 332. In Figure 7d and e corresponding to Complexes 3 and 4, respectively, Glu 332 plays a very important role with the longer chain (R3 = NH_2_) and with the NH of a pyrrole ring of the PPPy structure. The rest of the interactions are similar to those in previous cases.

**Figure 8:**
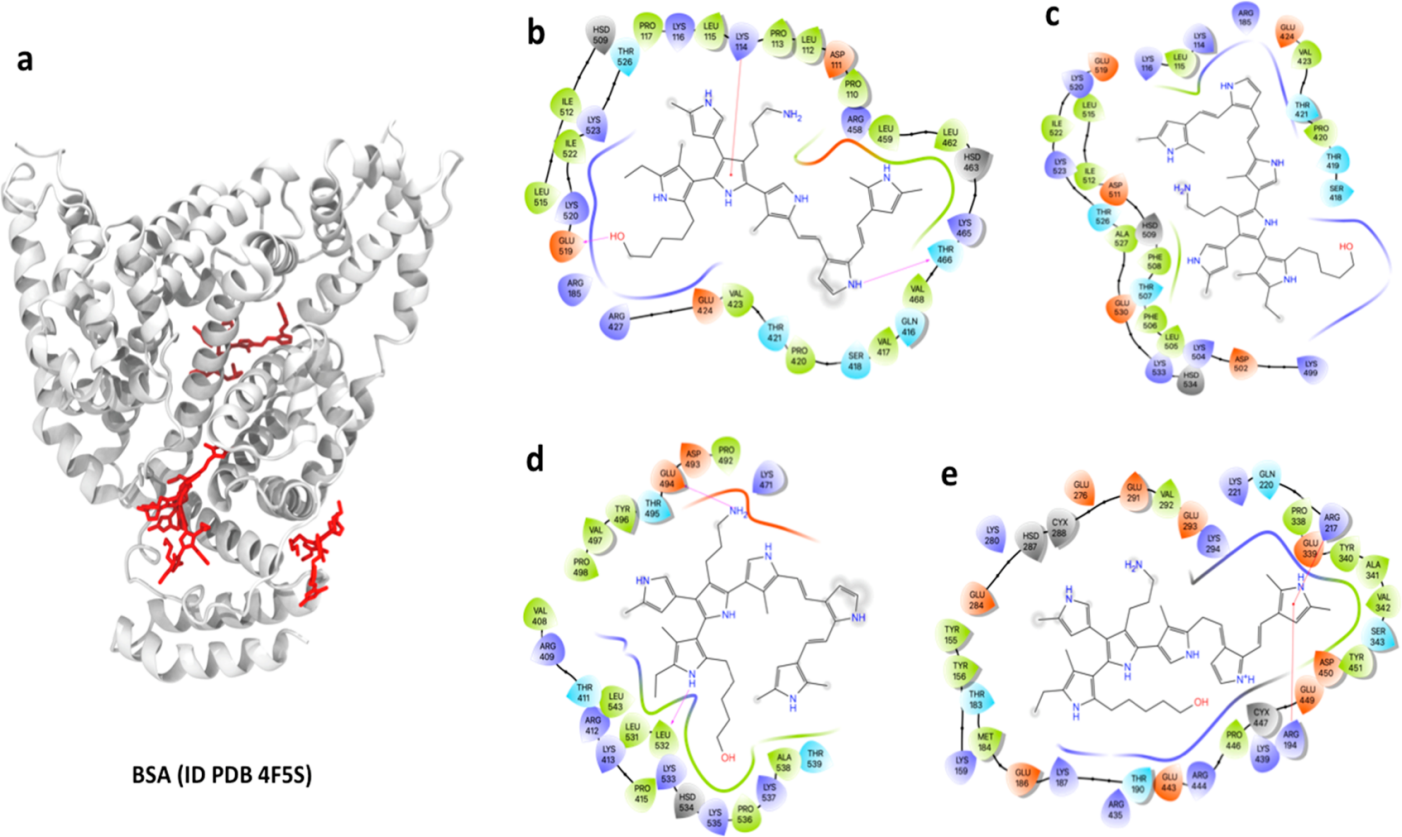
Best docked conformations for the complex of BSA with PPPy: (a) Binding modes of the combinatorial model R1 = NH_2_, R2 = CH_3_, and R3 = OH (red color) to BSA: Compounds were ordered by binding energy of the most populated cluster of each group according to Table 2 and prepared with VMD: (b) Group 1 (Complex 1), (c) Group 2 (Complex 1), (d) Group 3 (Complex 1), and (e) Group 4 (Complex 1). Interaction maps were obtained with the Maestro 2019-4 program.

For the combinatorial models R1 = NH_2_, R2 = CH_3_, and R3 = OH on the surface of BSA, we found four different binding sites by docking studies. These binding sites correspond to the most populated cluster and presented the most favorable Δ*G*_b_ (Table 2, Complex 1) in each group, as shown in Figure 8.

**Table 2:** Δ*G*_b_ Summary of BSA with the Combinatorial Model R1 = NH_2_, R2 = CH_3_, and R3 = OH of the PPPy structure determined at pH 7.2 by APBS and VMD1.9.1

Clustering analysis		Δ*G*_solv_ (kJ/mol)	Δ*G*_Coul_ (kJ/mol)	Δ*G*_nonelec_ (kJ/mol)	Δ*G*_b_^a^ (kJ/mol)
Group 1	Complex 1	86	–187	–25	–126
	Complex 2	89	–184	–26	–121
	Complex 3	83	–173	–25	–115
	Complex 4	93	–174	–25	–106
Group 2	Complex 1	68	–182	–23	–137
	Complex 2	87	–176	–23	–112
	Complex 3	79	–163	–24	–106
	Complex 4	81	–163	–23	–107
Group 3	Complex 1	54	–194	–22	–162
	Complex 2	57	–138	–23	–104
	Complex 3	62	–116	–21	–75
	Complex 4	53	–86	–20	–53
Group 4	Complex 1	64	–254	–25	–215
	Complex 2	61	–173	–23	–135

a Δ*G*_b_ = Δ*G*_solv_ + Δ*G*_Coul_ + Δ*G*_nonelec_ (*).

Our results showed four sites for the PPPy structure (combinatorial model R1 = NH_2_, R2 = CH_3_, and R3 = OH) on the surface of BSA (Figure 8a). Figure 8b shows the binding mode of the PPPy structure, where two hydrogen bonds were formed with BSA. A hydrogen bond presented the longest chain (R3 = OH) of the PPPy structure and the COO^–^ group of Glu 519 (O–H•••O, respectively). A π-cation-type interaction was also observed between the side chain of Lys 114 (NH_3_^+^) and a ring of the PPPy structure. Figure 8c depicts that the longest chain (R3 = OH) of the PPPy structure caused an electrostatic environment with Lys 499 and Lys 504, similar to R1 = NH_2_ with Asp 511 of BSA. As seen in Figure 8d, the longest chain (R3 = OH) established electrostatic interactions with Lys 533, Lys 535 and Lys 537, and the chain R1 = NH_2_ also presented electrostatic-type interactions with Asp 493 and Glu 494 (with this last amino acid forming a hydrogen bond). Figure 8e shows the π-cation interactions between the side chains of Asp 194 and Asp 217 with a ring of the PPPy structure, and the chain R1 = NH_2_ also established electrostatic interactions with Glu 276, Glu 291 and Glu 293 of BSA. However, the longest chain (R3 = OH) of the PPPy structure was not stretched. In all cases, the shortest chain (R2 = CH_3_) of the PPPy structure was close to hydrophobic amino acids.

#### Binding Energy (Δ*G*_b_) Calculations

The energetically favorable contributions to the Δ*G*_b_ for BSA with the combinatorial model R1 = OH, R2 = CH_3_, and R3 = NH_2_ of the PPPy structure are shown in Table 1, and we observe that the Δ*G*_b_ values were governed mainly by Coulombic interactions. Notably, in this combinatorial model, R1 = OH, R2 = CH_3_, and R3 = NH_2_, 1000 complexes were analyzed, and those that presented very favorable Δ*G*_b_ (from −350 to −415 kJ/mol) bound to the same site.

Finally, Table 2 shows the Δ*G*_b_ values of the second combinatorial model (R1 = NH_2_, R2 = CH_3_, R3 = OH) of the PPPy structure to BSA. In all groups, the computational results suggested that the binding process of BSA with the combinatorial model R1 = NH_2_, R2 = CH_3_, and R3 = OH of the PPPy structure was governed by electrostatic interactions, mainly by Coulombic interactions, and a minor proportion by hydrophobic interactions. However, Group 4 presented the most favorable Δ*G*_b_, since π-cations (Arg 194 and Arg 217, both with a ring of PPPy structure) and electrostatic interactions (between the R1= NH_2_ of the PPPy structure and three glutamic amino acids) were established in this binding site. As already described above, electrostatic interactions were involved in all groups, mainly due to the positions of R1 = NH_2_ and R3 = OH in the PPPy structure.

The Δ*G*_b_ values of the complexes were more favorable using the first combinatorial model R1 = OH R2 = CH_3_ R3 = NH_2_ compared to R1 = NH_2_, R2 = CH_3_, and R3 = OH of the PPPy structure to BSA. Additionally, the Coulombic component (Δ*G*_Coul_) became more favorable in the first combinatorial model, and Δ*G*_nonelec_ values were very close with respect to the second combinatorial model.

The binding free energy results are complementary with docking studies because the binding was more specific using the first combinatorial model with BSA, since four sites were found with the second combinatorial model. This implies that when the functional group NH_2_ is in the longest chain of the modified PPPy structure, it plays a very important role in the protein interaction and the binding becomes more specific.

## Conclusions

In this work, we studied the interaction between BSA and PPPy/I using experimental and *in silico* assays. The first part was performed using QCM studies, and we demonstrated that BSA exhibits a stronger attractive interaction with a PPPy surface than that with a bare gold surface. This is attributed mainly to electrostatic forces from the negatively charged BSA and the partially positive surface due to the protonation of NH_2_ at the studied pH. Also, the Langmuir equilibrium constant calculated indicates that there is high affinity to the surface compared to what it is observed in a gold surface.

Since the QCM experiments were not able to provide information on the phenomenon of molecular recognition with BSA, we proposed that the interaction was carried out with small fragments of the modified PPPy structure based on the structure reported by Kumar et al.^26^ as an “average characteristic surface”. In consequence, this structure allowed us to make combinations of functional groups that are present in the experimental PPPy surface as NH_2_, CH_3_ and OH.

Our BSA-PPPy simulation findings confirm that when the NH_2_ and OH groups are located in the longest chains (R1 and especially R3 positions) of the modified PPPy structure, electrostatic interactions are strongly favored in comparison with nonelectrostatic (Tables 1 and 2). In our case, the R2 position probably does not exert an effect on the interactions since it is a very short chain. However, it is important to mention that if the CH_3_ group is in the R3 position, it favors nonelectrostatic interactions with hydrophobic regions of BSA.

These experimental and computational results provide insight into the molecular recognition between PPPy and BSA, complementing various experiments carried out by our research group and establishing the importance of the electrostatic interactions between this polymer and protein. These results are important knowledge for different research groups and mainly complement what was reported by Fabela et al.,^5,6^ in which the functional recovery of the TSCI is dependent on the amount of BSA adsorbed on the PPPy surface.

## Supporting Information Available

DLS of the BSA protein prepared in phosphate buffer (pH 7.2) at 2 μM, physicochemical characterization of PPPy/I films, AFM images of different regions of the sensors of gold, and gold coated with PPPy/I with various magnification sizes, computational studies of human serum albumin (HSA) and PPPy combinatorial models (PDF)

## Abbreviations

AFM: atomic force microscopy
APBS: Adaptive Poisson–Boltzmann Solver
*C*: calibration constant
C ≡ N: nitrile
CH_3_: methyl
DLS: dynamic light scattering
Δ*f*: frequency shift
Δ*G*_ele_: electrostatic energy
Δ*G*_b_: free binding energy
Δ*G*_nonelec_: nonelectrostatic energy
Δ*G*_solv_: solvation energy
Δ*G*_Coul_: Coulombic energies
ΔSASA: solvent-accessible surface area change
ΔΓ: surface coverage
*E*_loss_: energy dissipated
*E*_stored_: stored energy
γ: HSA, BSA, human and bovine serum albumin interfacial tension coefficient
*K*: adsorption constant
*n*: is the overtone order, which normalizes the equation
NH_2_: amino
OH: hydroxyl
PPPy/I: plasma polymerized pyrrole/iodine
PDB: Protein Data Bank
QCM: quartz crystal microbalance
RSMD: root-mean-square deviation
TSCI: traumatic spinal cord injury
VMD: Visual Molecular Dynamics
