# Supplementary material for: Quartz Crystal Microbalance Application and In Silico Studies to Characterize the Interaction of Bovine Serum Albumin with Plasma Polymerized Pyrrole Surfaces: Implications for the Development of Biomaterials
Source: Langmuir. 2023 Aug 1;39(32):11213–23. doi: 10.1021/acs.langmuir.3c00308 (PMC10921548; doi:10.1021/acs.langmuir.3c00308)
Supplement: Supplementary file 1 — la3c00308_si_001.pdf [file la3c00308_si_001.pdf]

Supporting Information

Quartz crystal microbalance application and *in silico* studies to characterize the interaction of bovine serum albumin with plasma polymerized pyrrole surfaces: implications for the development of biomaterials

Iris N. Serratos[a], Alberto S. Luviano[b, †] , Cesar Millan-Pacheco[c], Juan Morales-Corona[d], Estephanny Jocelyn Alvarado Muñoz[e], José Campos-Terán[f,*], Roberto Olayo[d*]

[a]Departamento de Química, Universidad Autónoma Metropolitana-Iztapalapa, Ciudad de México, 09340, México.
[b]Laboratorio de Biofisicoquímica, Departamento de Fisicoquímica, Facultad de Química, Universidad Nacional Autónoma de México, Ciudad de México, 04510, México.
[c]Facultad de Farmacia, Universidad Autónoma del Estado de Morelos, Morelos, 62209, México.
[d]Departamento de Física, Universidad Autónoma Metropolitana-Iztapalapa, Ciudad de México, 09340, México.
[e]Departamento de Ingeniería Eléctrica, Universidad Autónoma Metropolitana-Iztapalapa, Ciudad de México, 09340, México.
[f]Departamento de Procesos y Tecnología, Universidad Autónoma Metropolitana-Cuajimalpa, Ciudad de México, 05348, México.

*Corresponding authors:
E-mail address: jcampos@cua.uam.mx (J. Campos-Terán), oagr@xanum.uam.mx (R. Olayo)

Keywords: Plasma polymerized pyrrole surfaces (PPPy), bovine serum albumin (BSA), quartz crystal microbalance (QCM), adsorption constant, atomic force microscopy (AFM), *in silico* studies

1. Dynamic light scattering of BSA protein

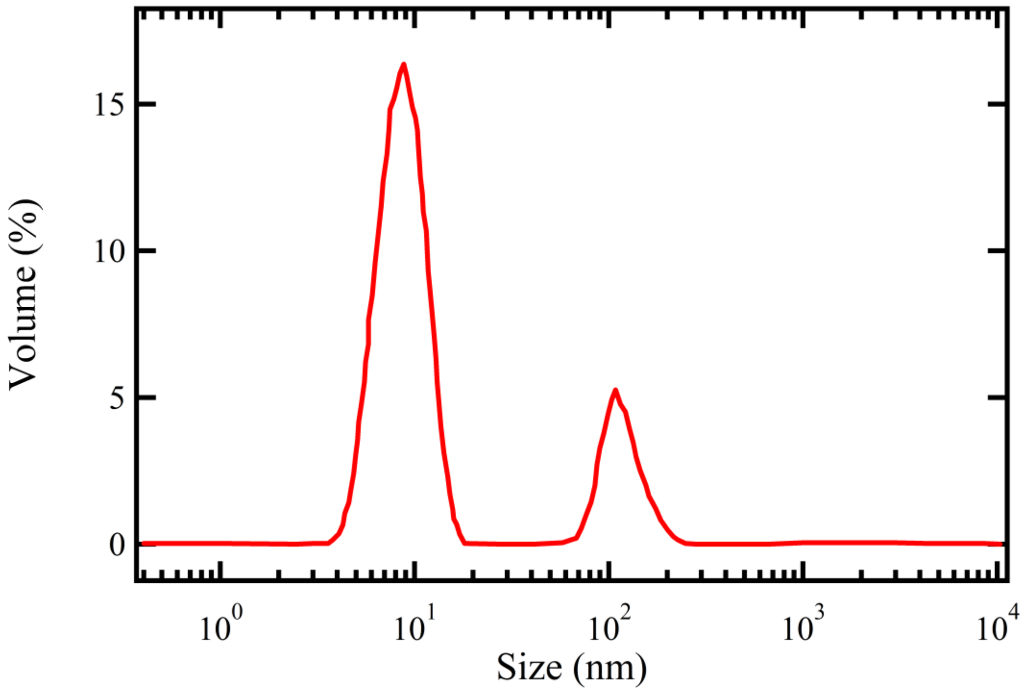

Figure S1. DLS from the BSA protein.

2. Physicochemical characterization of PPPy/I films

Plasma polymerized polypyrrole (PPPy) is a complex polymer with average characteristics that are reproducible, although its composition might change depending on the synthesis variables (power, pressure, reaction time). The synthesis conditions used in this work have already been reported.[1] for convenience we add this additional summary with the basic molecular and surface characterization: Fourier Transform Infrared (FTIR), X-ray Photonic Spectroscopy (XPS) and Contact Angle (CA).

FTIR spectrum

Figure S2 presents the FTIR spectrum of the PPPy/I, the region between 3500 and 3300 cm^{-1} shows a broad band with the NH and OH groups. In the case of nitrogen band, the group can belong to primary or secondary amines. Between 2960 and 2850 cm^{-1} we have alymphatic CH. In the frequency interval 2150-2260 cm^{-1} there is a band that can be assigned to vibrations of C≡N and C≡C. These last two bands are not found in the chemically synthesized polypyrrole, which gives evidence of monomer cleavage during plasma polymerization. In 1593 cm^{-1} we have a band that can be associated with amine groups and C=C groups associated with the pyrrole ring, in 1495 we have the C-N groups, this resonance as well as that of 1250 cm^{-1} appear at these frequencies because they are associated with the pyrrole aromatic ring.

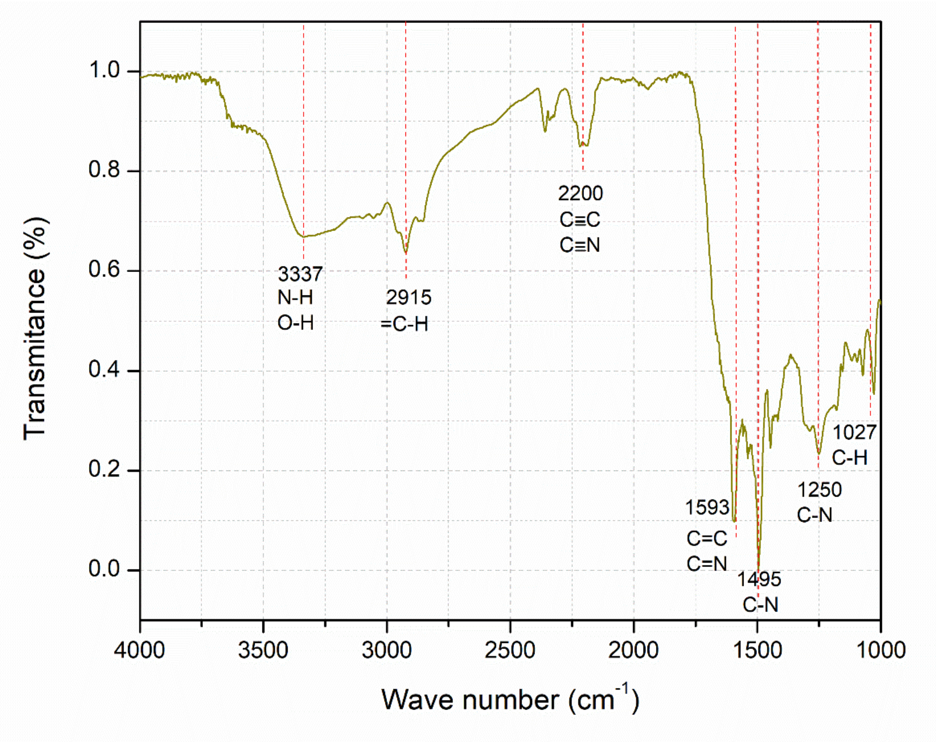

Figure S2. A1 Infrared spectrum of the PPPy/I.

The contact angle of the PPPy/I surface is 82±3, and the XPS spectrum is shown in Figure S3. The signal corresponding to C 1 s is centered at 285.0 eV with an area of 72.9%, O 1s has an area of 16.3%, N 1s has an area of 10.8%. The composition of the polymer has been discussed in detail in a previous work.[1,2]

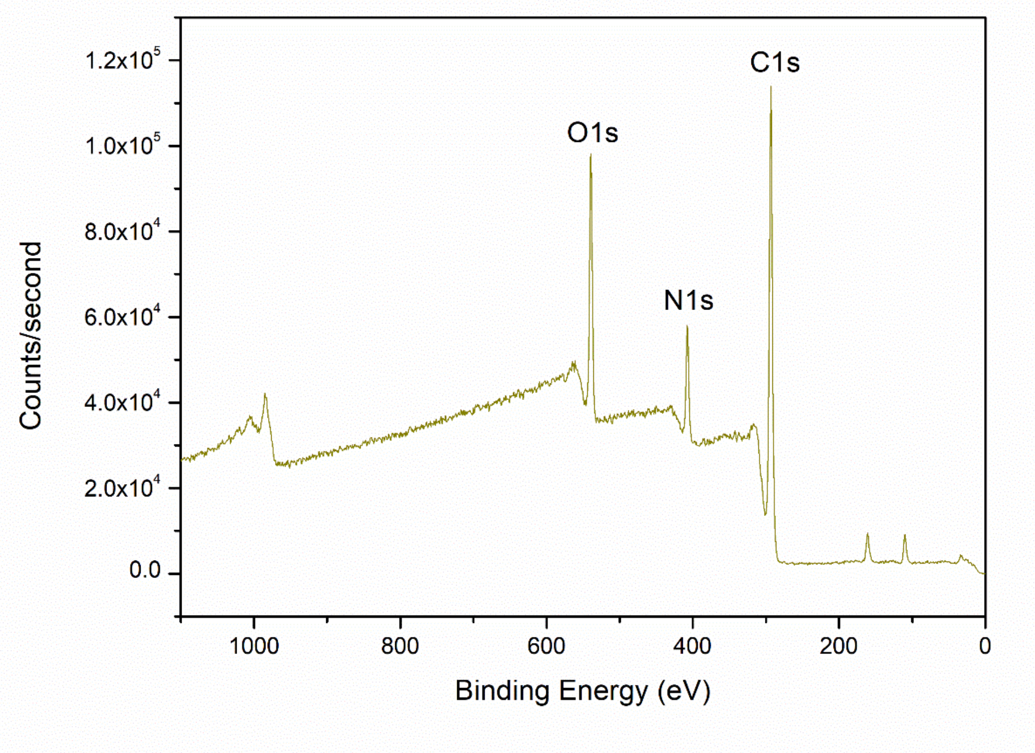

Figure S3. XPS spectrum of PPPy/I.

3. Atomic force microscopy images

Figure S4. The PPPy/I surface was analyze in three regions.

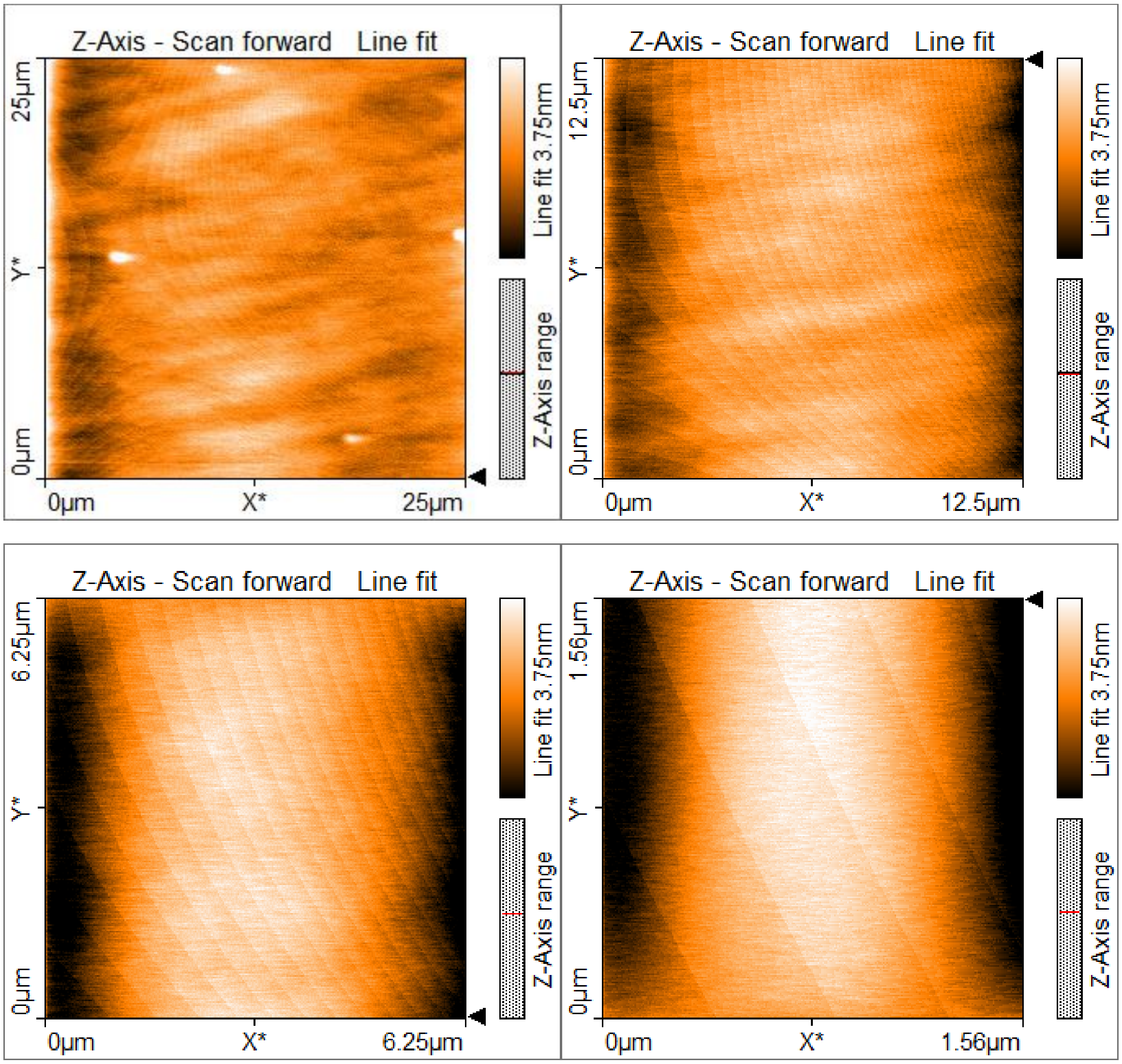

Region 1. AFM images at different scales, clearly shows the pattern acquired from the electromagnetic field.

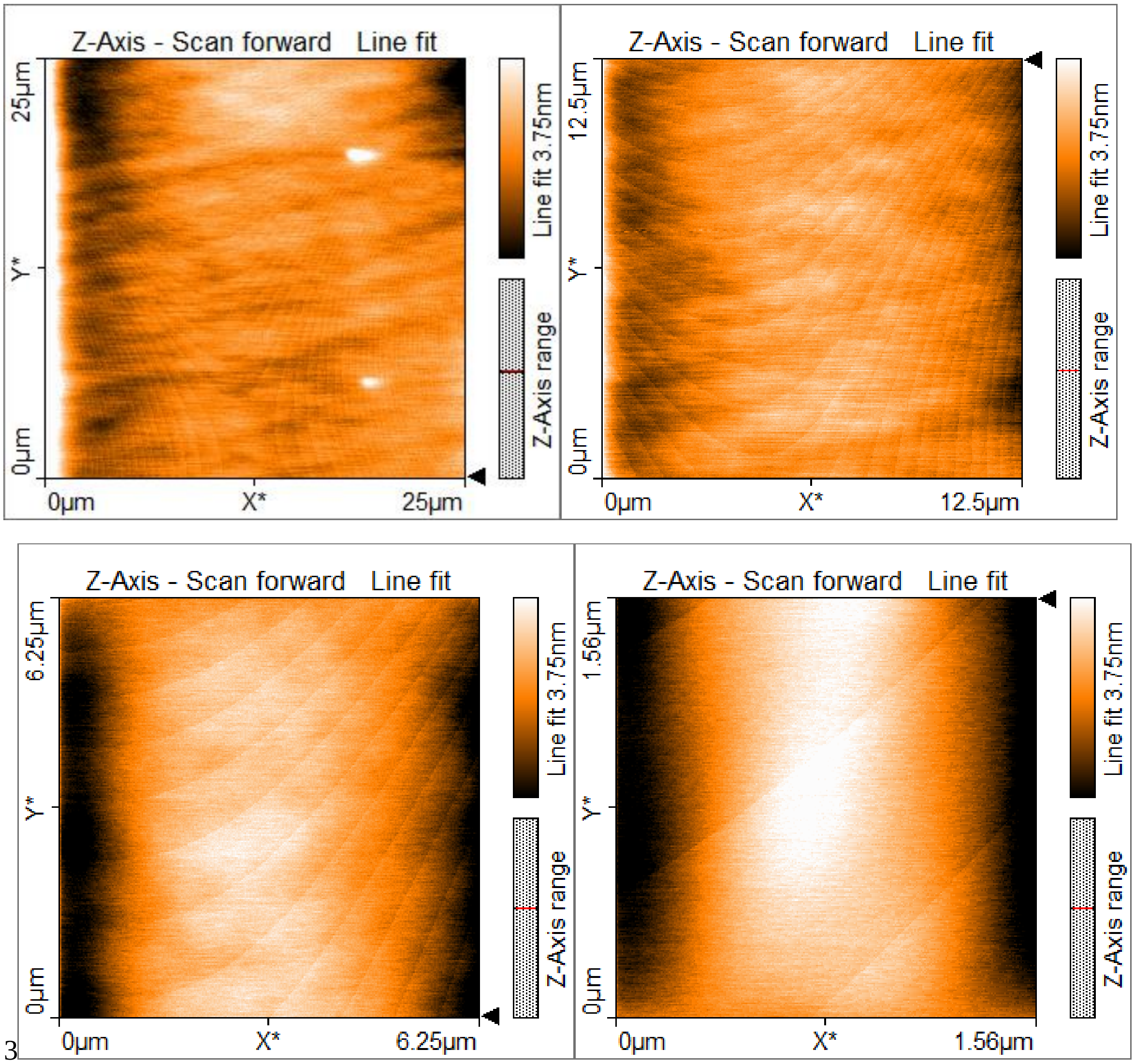

3

Region 2. AFM images at different scales, clearly shows the pattern acquired from the electromagnetic field.

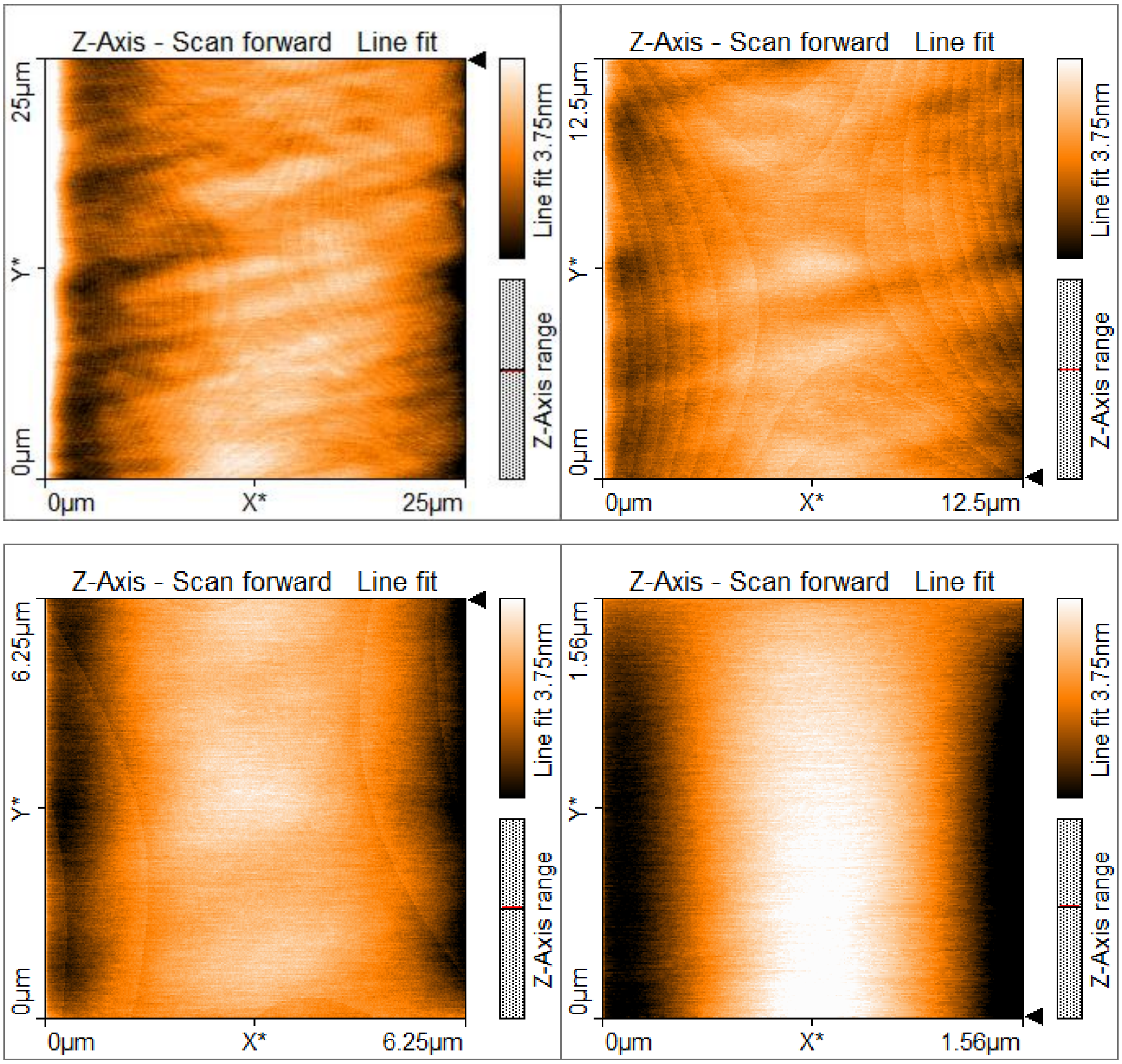

Region 3. AFM images at different scales, clearly shows the pattern acquired from the electromagnetic field.

Figure S5. Surfaces of clean gold, PPPy/I, and PPPy/I after protein adsorption were analyzed using AFM technique from different regions at different scales.

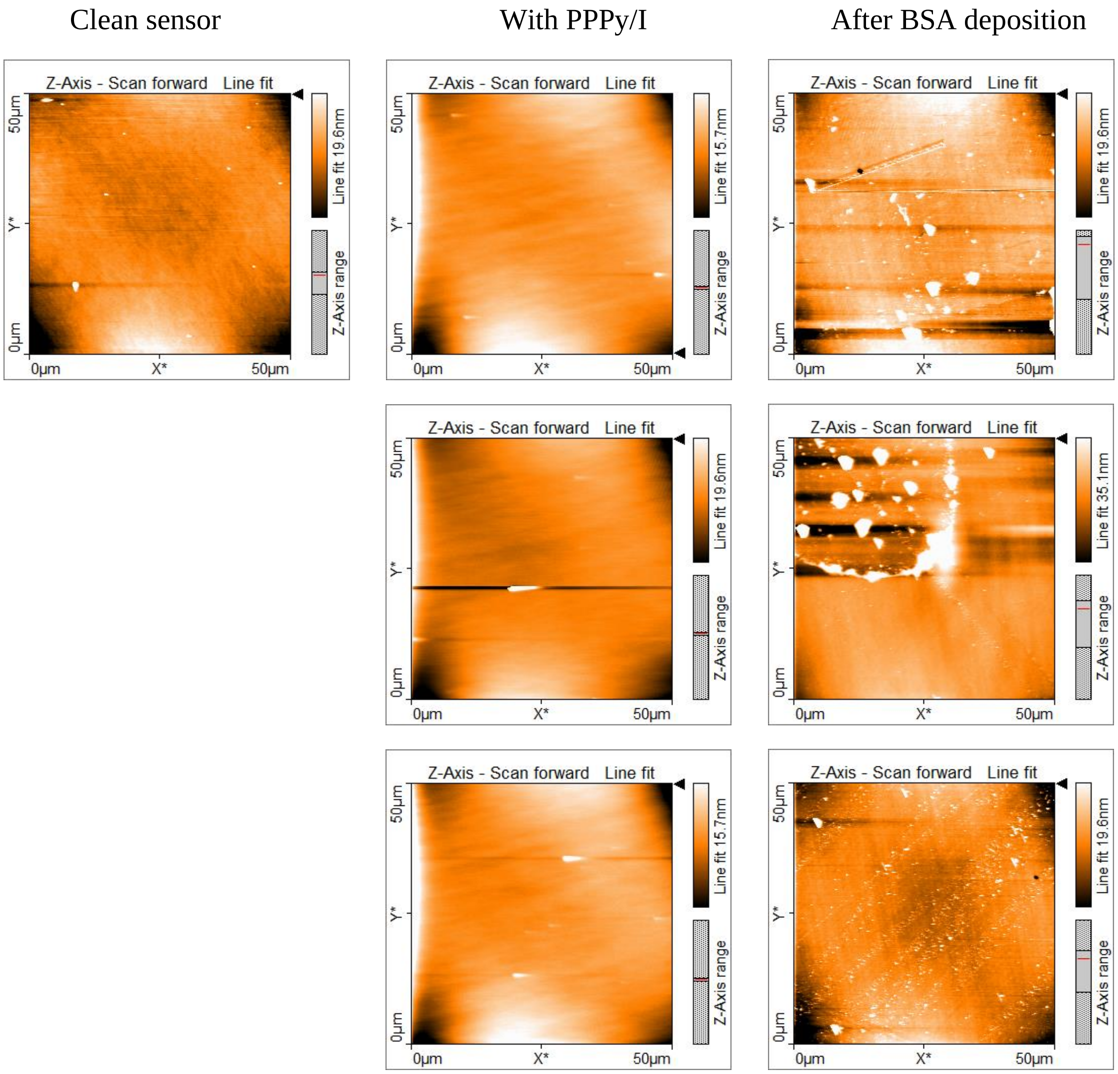

Figure S5a. Images of 50×50 μm^2 from clean gold sensor in one region, and with PPPy before and after protein adsorption in three regions.

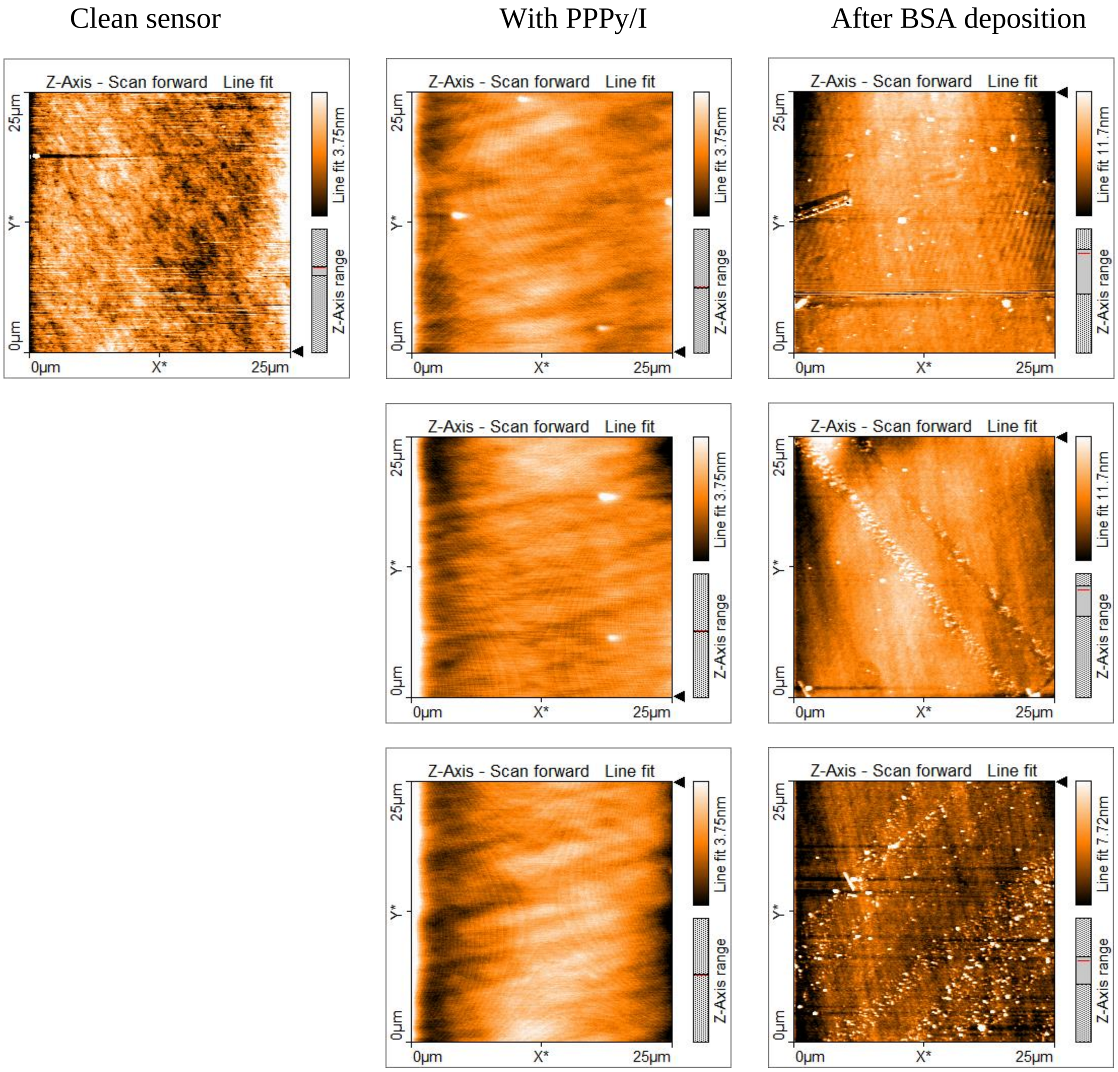

Figure S5b. Images of 25×25 µm^2 from clean gold sensor in one region, and with PPPy before and after protein adsorption in three regions.

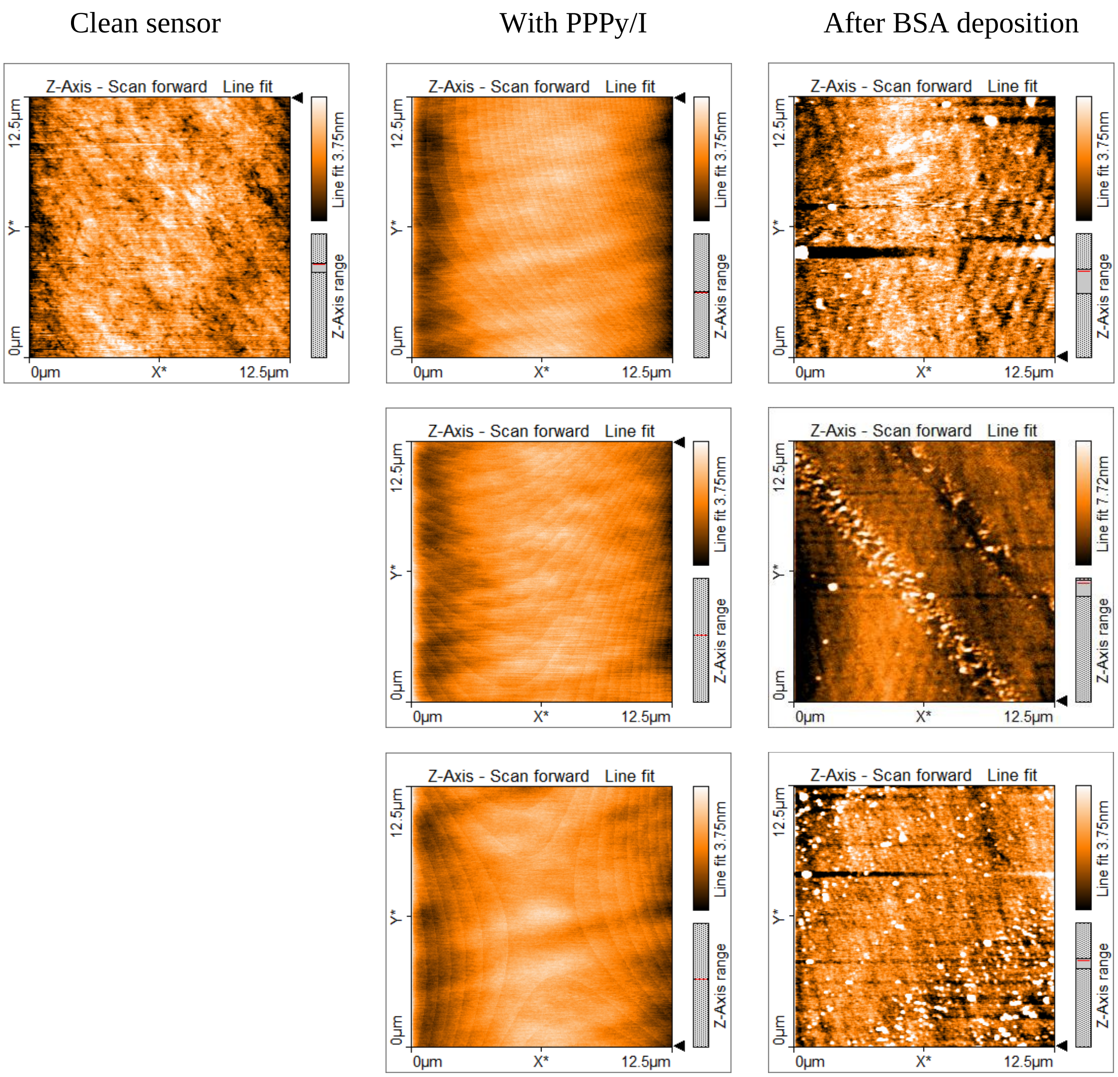

Figure S5c. Images of 12.5×12.5 μm^2 from clean gold sensor in one region, and with PPPy before and after protein adsorption in three regions.

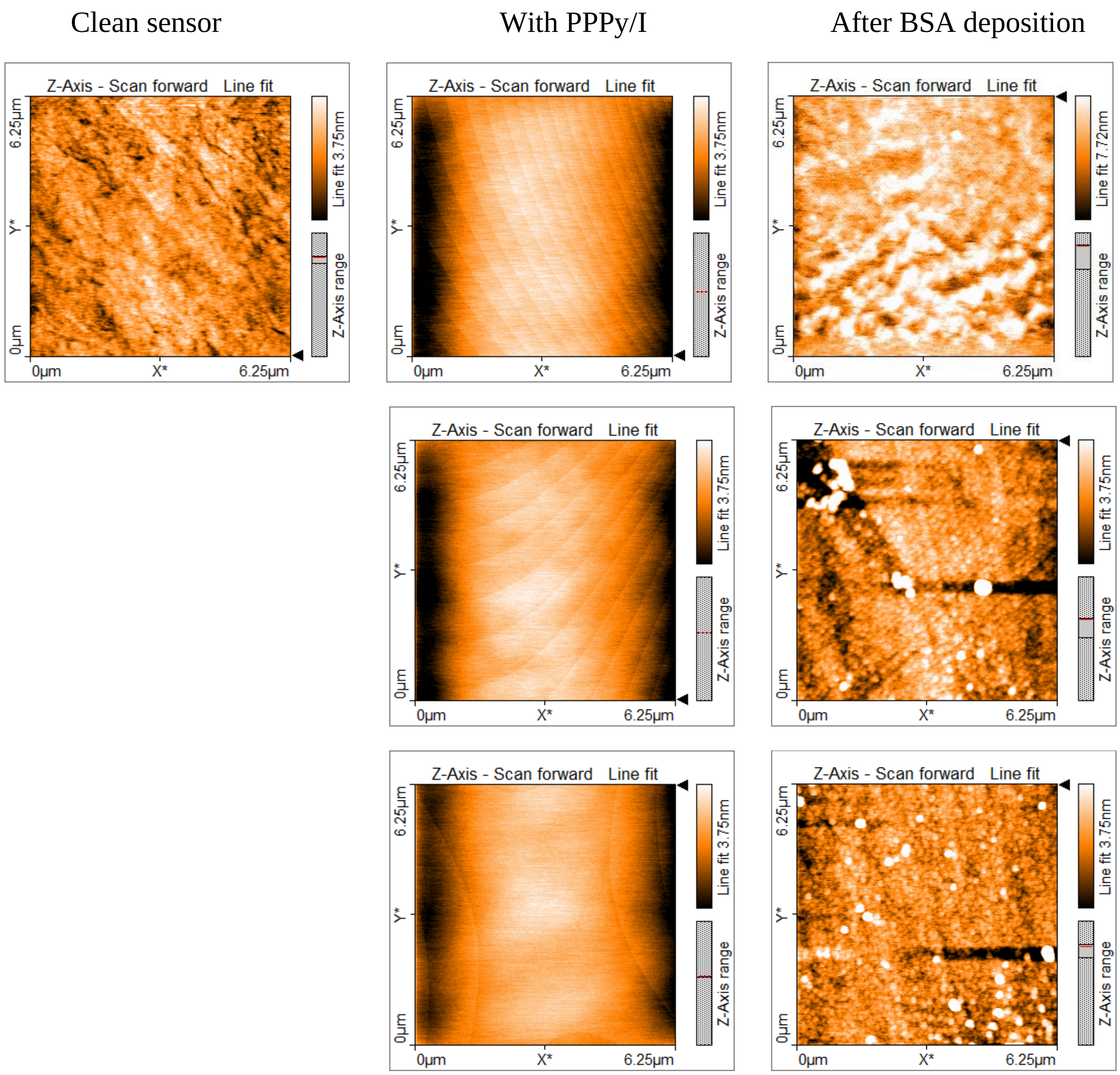

Figure S5d. Images of 6.25×6.25 μm^2 from clean gold sensor in one region, and with PPPy before and after protein adsorption in three regions.

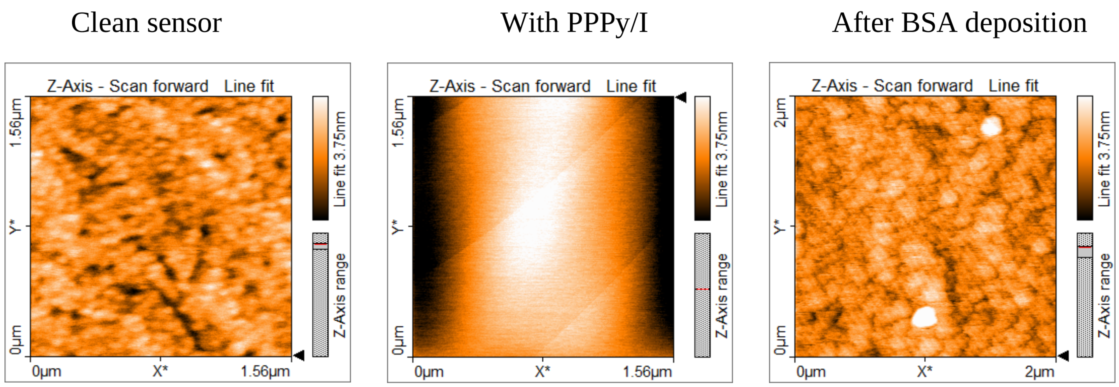

Figure S5e. Images of 1.56×1.56 μm^2 from clean gold sensor, and with PPPy before and after protein adsorption in one region.

4. Computational studies of human serum albumin (HSA) and PPPy combinatorial models.

Figure S6. Exhaustive study of molecular dynamics simulations of HSA and its interaction with PPPy combinatorial models by molecular docking assays and binding energy calculations.

Human albumin (PDB ID 5YB1)[3] was used as initial structure for molecular dynamics simulations. Albumin structure was prepared on CHARMM-GUI server.[4–6] All hydrogens were used and a 0.15 mM of KCl concentration was used. Periodic Boundary conditions were placed and PME for electrostatics were used as indicated on CHARMM-GUI server. GROMACS input was used. CHARMM 36m potential[7,8] was used on GROMACS 2019.[9–11] A 2 fs integration time step was used for 100 ns. Three independent 100 ns molecular dynamics simulations were conducted. Root mean square deviation, distances and cluster analysis were conducted with the tools included on GROMACS.

Molecular docking was conducted using Autodock Vina.[12] 100 independent blind molecular docking experiments were conducted on each cluster structure (one for each system). All outputs were root mean square deviations (RMSD) clustered and the most populated one is herein presented. Images were produced using VMD 1.9.3[13] and Chimera UCSF.[14,15]

We conducted three independent 100 ns molecular dynamics simulations on human albumin. Figure S6.1A illustrates the temporal fluctuations observed in the α-carbon RMSD for the entire protein over the 100 ns simulated against the initial structure. As noted, all systems appeared to be fluctuating along the simulated time. To pinpoint the specific region responsible for these RMSD fluctuations, we focused our analysis on the final 40 ns of each simulation. Upon analyzing residues 3 to 300 (Figure S6.1B on blue) and 301 to 583 (Figure S6.1B on orange), we discovered that both sections exhibited remarkable stability throughout the analyzed time (Figure S6.1C).

Consequently, we concluded that the molecular dynamics simulations remained stable during the last 40 ns, and the observed RMSD fluctuations were attributed to movements in both sections of the protein.

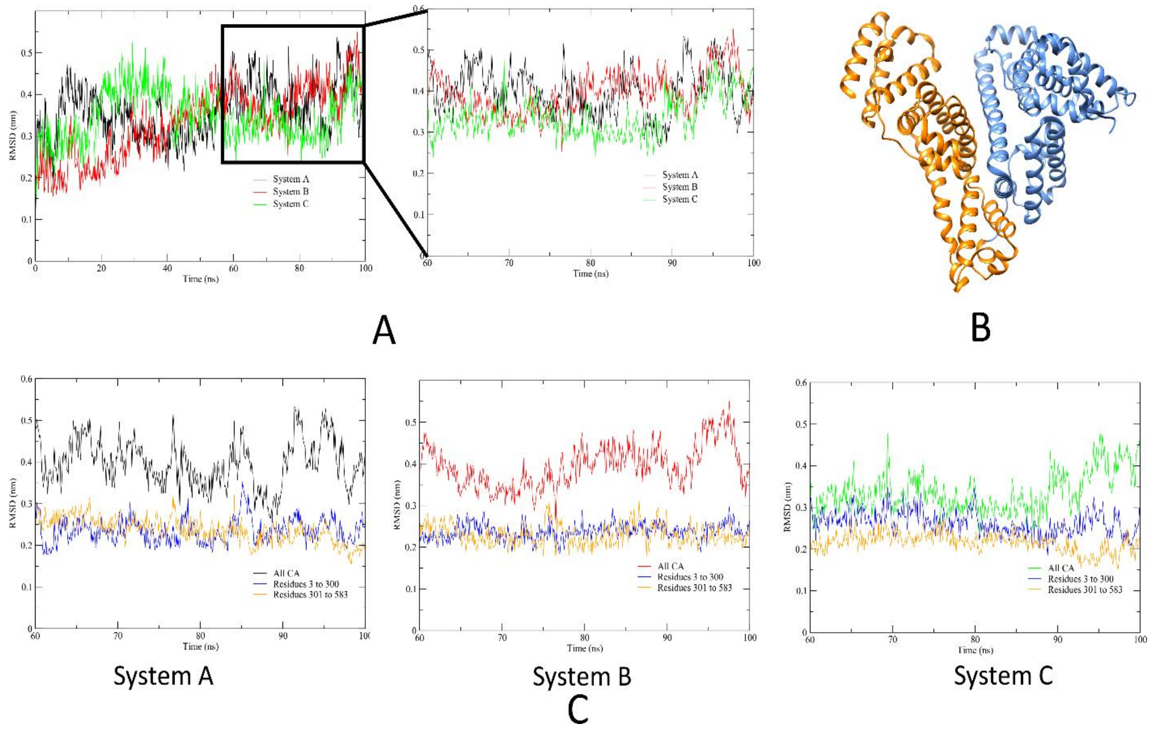

Figure S6.1. A) α-carbons RMSD over 100 ns of each system simulated. B) Albumin segments used to calculate the RMSD (blue, residue 3 to 300 and orange, residues 301-583). C) RMSD over the last 40 ns of each system showing the RMSD for all the system and by segment.

To determine if the RMSD fluctuations earlier were due to domain movements (like tweezers), we calculated the distance between the center of mass (COM) of each domain (3-300 and 301-583), Figure S6.2. As noted, all systems initiated with a distance of around 3.24 nm and they diverged (as expected due as they are independent simulations) along the time. The COM distance open and close along the time. Moreover, at the last 40 ns the distances on the three simulations converged to a similar value around 0.3 nm.

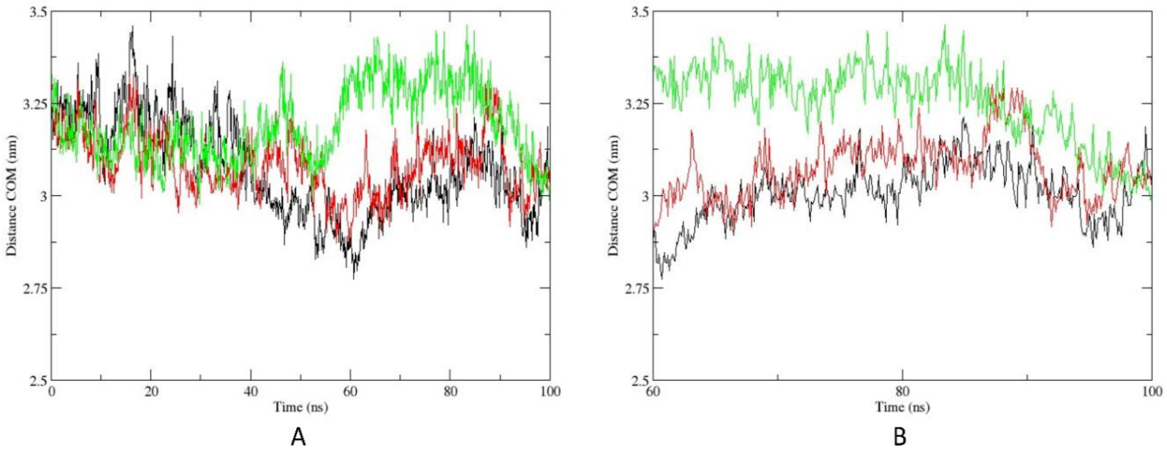

Figure S6.2. Distance of the center of mass of the two domains herein used (3-300 and 301-583). Color code are the same as Figure S6.1A (System A on black, B on red and C on green).

Based on the results obtained, we look for a representative structure of the last 40 ns of each simulated system. A representative structure (the most populated cluster contains at least 70% of the structures analyzed) of the last 40 ns were obtained for each system. Figure S6.3 shows the superposition between each cluster found against the initial structure.

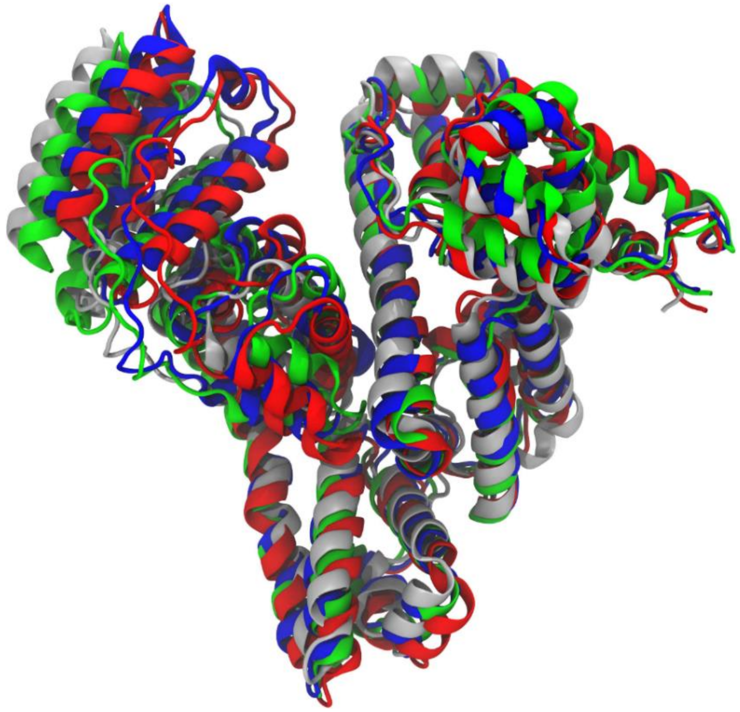

Figure S6.3. Cartoon representation of each cluster found for each system (System A on blue, B on red and C on green) against initial structure.

Each structure was used as receptor for a blind docking with modified PPPy ligand with two combinatorial compounds CC 1 (R1=OH, R2=CH_3, R3=NH_2); and CC 2 (R1=NH_2,

$R2=CH_3$, R3=OH). Molecular affinity energies for the most populated cluster are shown in Table S1.

Table S1. Affinities Energies between each cluster found from albumin molecular dynamics and combinatorial compounds (CC).

	Affinity energy (kcal/mol)
Cluster A/CC 1	-8.24 +/- 0.59
Cluster B/CC 1	-7.55 +/- 0.50
Cluster C/CC 1	-8.17 +/- 0.37
Cluster A/CC 2	-7.85 +/- 0.68
Cluster B/CC 2	-7.85 +/- 0.43
Cluster C/CC 2	-8.29 +/- 0.31

As noted from Table S1, all affinities energies were similar. These results showed that CC 1 and CC 2 may bound albumin with similar affinities independent of the protein conformation. To determine the position of both combinatorial compounds on the albumin surface we obtained the interaction maps between the ligands and the protein. Interaction maps are found in Figure S6.4.

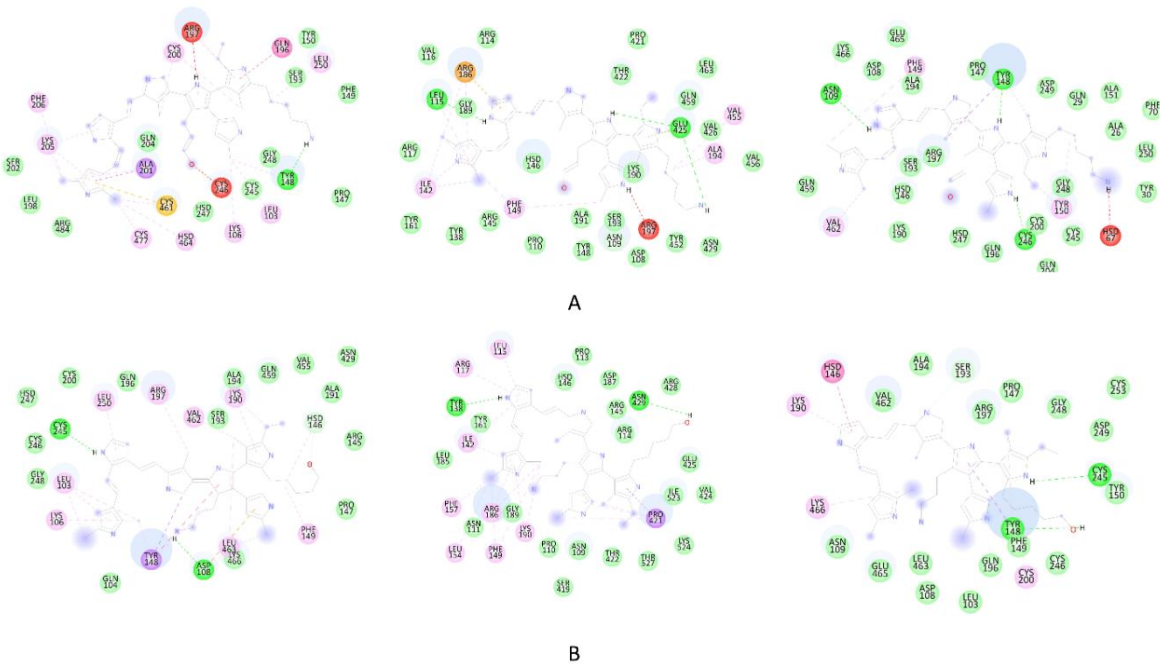

Figure S6.4. Interaction maps between Combinatorial Compound 1 and 2 against each cluster found from molecular dynamics. Cluster A (left), B (center) and C (right) for A) Combinatorial Compound A and B) Combinatorial Compound B.

Subsequently, we calculated the ΔG_b for each combinatorial model of the modified PPPy structure on the representative structure of HSA of the most populated clusters of each molecular dynamics, we considered the electrostatic and non-electrostatic components as described by Baker et al.[16]

Table S2. ΔG_b values for the representative structure of HSA of the most populated clusters of each molecular dynamics with the CC 1 (R1=OH R2=CH_3, R3=NH_2) of the modified PPPy structure determined at pH 7.2 by APBS and VMD1.9.1.

Clustering analysis	ΔG_{solv} (kJ/mol)	ΔG_{Coul} (kJ/mol)	$\Delta G_{non-elec}$ (kJ/mol)	$\Delta G_b{}^a$ (kJ/mol)
Cluster A/CC 1	93	-154	-25	-86
Cluster B/CC 1	124	-136	-30	-42
Cluster C/CC 1	94	18	-29	86

$* \Delta G_b = \Delta G_{solv} + \Delta G_{Coul} + \Delta G_{non-elec}$

Table S3. ΔG_b values for the representative structure of HSA of the most populated clusters of each molecular dynamics with the CC 2 (R1=NH_2, R2=CH_3, R3=OH) of the modified PPPy structure determined at pH 7.2 by APBS and VMD1.9.1.

Clustering analysis	ΔG_{solv} (kJ/mol)	ΔG_{Coul} (kJ/mol)	$\Delta G_{non-elec}$ (kJ/mol)	$\Delta G_b{}^a$ (kJ/mol)
Cluster A/CC 2	89	-123	-29	-63
Cluster B/CC 2	88	-81	-31	-24
Cluster C/CC 2	58	11	-27	42

$* \Delta G_b = \Delta G_{solv} + \Delta G_{Coul} + \Delta G_{non-elec}$

As observed, the HSA ΔG_b values changed in all the molecular dynamics simulations. However, we showed that the trend in both HSA and BSA was preserved. As with BSA, the HSA interaction for CC 1 (R1=OH, R2=CH_3, R3=NH_2) of the modified PPPy structure, the ΔG_b values were more favorable compared to CC 2 (R1=NH_2, R2=CH_3 and R3=OH). Furthermore, the binding is governed by the Coulombic component in both cases (HSA and BSA).

Also, the binding free energy results are complementary with docking studies because CC 1 established more hydrogen bonds compared to CC 2 mainly when the functional group NH_2 is in the longest chain of the modified PPPy structure compared to the group OH, and this aspect plays a very important role in the protein interaction, which in consequence was reflected in the ΔG_b values. On the other hand, in the Cluster C both CC 1 and CC 2 established hydrophobic interactions, hence the electrostatic component was not favored and therefore the $\Delta G>0$ values.

References

(1) Vasquez-Ortega, M.; Ortega, M.; Morales, J.; Olayo, M. G.; Cruz, G. J.; Olayo, R. Core-Shell Polypyrrole Nanoparticles Obtained by Atmospheric Pressure Plasma Polymerization. *Polym. Int.* **2014**, *63* (12), 2023–2029. https://doi.org/10.1002/pi.4756.

(2) Serratos, I. N.; Olayo, R.; Millán-Pacheco, C.; Morales-Corona, J.; Vicente-Escobar, J. O.; Soto-Estrada, A. M.; Córdoba-Herrera, J. G.; Uribe, O.; Gómez-Quintero, T.; Arroyo-Ornelas, M. Á.; Godínez-Fernández, R. Modeling Integrin and Plasma-Polymerized Pyrrole Interactions: Chemical Diversity Relevance for Cell Regeneration. *Sci. Rep.* **2019**, *9* (1), 1–12. https://doi.org/10.1038/s41598-019-43286-4.

(3) Wang, J.; Gou, Y.; Zhang, Z.; Yu, P.; Qi, J.; Qin, Q.; Sun, H.; Wu, X.; Liang, H.; Yang, F. Developing an Anticancer Copper(II) Multitarget Pro-Drug Based on the His146 Residue in the IB Subdomain of Modified Human Serum Albumin. *Mol. Pharm.* **2018**, *15* (6), 2180–2193. https://doi.org/10.1021/acs.molpharmaceut.8b00045.

(4) Brooks, B. R.; Brooks, C. L.; Mackerell, A. D.; Nilsson, L.; Petrella, R. J.; Roux, B.; Won, Y.; Archontis, G.; Bartels, C.; Boresch, S.; Caflisch, A.; Caves, L.; Cui, Q.; Dinner, A. R.; Feig, M.; Fischer, S.; Gao, J.; Hodoscek, M.; Im, W.; Kuczera, K.; Lazaridis, T.; Ma, J.; Ovchinnikov, V.; Paci, E.; Pastor, R. W.; Post, C. B.; Pu, J. Z.; Schaefer, M.; Tidor, B.; Venable, R. M.; Woodcock, H. L.; Wu, X.; Yang, W.; York, D. M.; Karplus, M. CHARMM: The Biomolecular Simulation Program. *J. Comput. Chem.* **2009**, *30* (10), 1545–1614. https://doi.org/10.1002/jcc.21287.

(5) Jo, S.; Kim, T.; Iyer, V. G.; Im, W. CHARMM-GUI: A Web-Based Graphical User Interface for CHARMM. *J. Comput. Chem.* **2008**, *29* (11), 1859–1865. https://doi.org/10.1002/jcc.20945.

(6) Lee, J.; Cheng, X.; Swails, J. M.; Yeom, M. S.; Eastman, P. K.; Lemkul, J. A.; Wei, S.; Buckner, J.; Jeong, J. C.; Qi, Y.; Jo, S.; Pande, V. S.; Case, D. A.; Brooks, C. L.; MacKerell, A. D.; Klauda, J. B.; Im, W. CHARMM-GUI Input Generator for NAMD, GROMACS, AMBER, OpenMM, and CHARMM/OpenMM Simulations Using the CHARMM36 Additive Force Field. *J. Chem. Theory Comput.* **2016**, *12* (1), 405–413. https://doi.org/10.1021/acs.jctc.5b00935.

(7) Huang, J.; Rauscher, S.; Nawrocki, G.; Ran, T.; Feig, M.; de Groot, B. L.; Grubmüller, H.; MacKerell, A. D. CHARMM36m: An Improved Force Field for Folded and Intrinsically Disordered Proteins. *Nat. Methods* **2017**, *14* (1), 71–73. https://doi.org/10.1038/nmeth.4067.

(8) Huang, J.; MacKerell, A. D. CHARMM36 All-Atom Additive Protein Force Field: Validation Based on Comparison to NMR Data. *J. Comput. Chem.* **2013**, *34* (25), 2135–2145. https://doi.org/10.1002/jcc.23354.

(9) Abraham, M. J.; Murtola, T.; Schulz, R.; Páll, S.; Smith, J. C.; Hess, B.; Lindahl, E.

GROMACS: High Performance Molecular Simulations through Multi-Level Parallelism from Laptops to Supercomputers. *SoftwareX* **2015**, *1–2*, 19–25. https://doi.org/10.1016/j.softx.2015.06.001.

(10) Berendsen, H. J. C.; van der Spoel, D.; van Drunen, R. GROMACS: A Message-Passing Parallel Molecular Dynamics Implementation. *Comput. Phys. Commun.* **1995**, *91* (1–3), 43–56. https://doi.org/10.1016/0010-4655(95)00042-E.

(11) Van Der Spoel, D.; Lindahl, E.; Hess, B.; Groenhof, G.; Mark, A. E.; Berendsen, H. J. C. GROMACS: Fast, Flexible, and Free. *J. Comput. Chem.* **2005**, *26* (16), 1701–1718. https://doi.org/10.1002/jcc.20291.

(12) Trott, O.; Olson, A. J. AutoDock Vina: Improving the Speed and Accuracy of Docking with a New Scoring Function, Efficient Optimization, and Multithreading. *J. Comput. Chem.* **2009**, 455–461. https://doi.org/10.1002/jcc.21334.

(13) Humphrey, W.; Dalke, A.; Schulten, K. VMD: Visual Molecular Dynamics. *J. Mol. Graph.* **1996**, *14* (1), 33–38. https://doi.org/10.1016/0263-7855(96)00018-5.

(14) Meng, E. C.; Pettersen, E. F.; Couch, G. S.; Huang, C. C.; Ferrin, T. E. Tools for Integrated Sequence-Structure Analysis with UCSF Chimera. *BMC Bioinformatics* **2006**, *7* (1), 339. https://doi.org/10.1186/1471-2105-7-339.

(15) Pettersen, E. F.; Goddard, T. D.; Huang, C. C.; Couch, G. S.; Greenblatt, D. M.; Meng, E. C.; Ferrin, T. E. UCSF Chimera?A Visualization System for Exploratory Research and Analysis. *J. Comput. Chem.* **2004**, *25* (13), 1605–1612. https://doi.org/10.1002/jcc.20084.

(16) Baker, N. A.; Sept, D.; Joseph, S.; Holst, M. J.; McCammon, J. A. Electrostatics of Nanosystems: Application to Microtubules and the Ribosome. *Proc. Natl. Acad. Sci.* **2001**, *98* (18), 10037–10041. https://doi.org/10.1073/pnas.181342398.
